# Physical activity is associated with lower mortality in adults with obesity: a systematic review with meta-analysis

**DOI:** 10.1186/s12889-024-19383-z

**Published:** 2024-07-12

**Authors:** Vicente Martínez-Vizcaíno, Rubén Fernández-Rodríguez, Sara Reina-Gutiérrez, Eva Rodríguez-Gutiérrez, Miriam Garrido-Miguel, Sergio Núñez de Arenas-Arroyo, Ana Torres-Costoso

**Affiliations:** 1 Health and Social Research Center, Universidad de Castilla-La Mancha, Cuenca, Spain; 2 Faculty of Health Sciences, Universidad Autónoma de Chile, Talca, Chile; 3 Research Network On Chronicity, Primary Care and Health Promotion (RICAPPS), Cuenca, Spain; 4 Faculty of Physiotherapy and Nursing, Universidad de Castilla-La Mancha, Toledo, Spain; 5 Faculty of Nursing, Universidad de Castilla-La Mancha, Albacete, Spain

**Keywords:** Mortality, Cancer, Physical activity, Obesity

## Abstract

**Background:**

Obesity is a complex chronic disease associated with several adverse health outcomes that increase mortality risk. Physical activity (PA) is recommended for the prevention and treatment of obesity and is related to a decreased risk of cardiovascular disease, cancer and all-cause mortality. This systematic review and meta-analysis estimates the effect of PA levels on mortality (cardiovascular, cancer and all-cause mortality) in adults with obesity.

**Methods:**

A systematic search was conducted in MEDLINE, Embase, Web of Science and SPORTDiscus from inception to June 2024. Prospective cohort studies that explored the association between PA and mortality in adults with obesity (according to their body mass index, ≥ 30 kg/m^2^) aged ≥ 18 years were included. Our main outcomes were all-cause mortality, and cardiovascular, and cancer mortality reported in primary studies by hazard ratios or relative risk, which were pooled for the meta-analysis when at least two studies reported the effect estimate for the same outcome. The PRISMA recommendations and the MOOSE guidelines were followed. The reported mortality risk estimates comparing insufficiently active versus active (moderate to very active) adults with obesity were pooled using the DerSimonian and Laird random-effects model.

**Results:**

A total of 9 prospective cohort studies involving 199,425 adults with obesity (age range: 35–85 years) were included, of which 59,873 were insufficiently active and 84,328 were active. Active individuals had a 21% lower risk of all-cause mortality (HR: 0.79, 95%CI: 0.74 to 0.84; I^2^ = 38.2%), and a 24% lower risk of cardiovascular mortality (HR: 0.76, 95%CI: 0.66 to 0.87; I^2^ = 0.0%) than insufficiently active individuals. The HR for cancer mortality was 0.91 (95%CI: 0.80 to 1.02; I2 = 0.0%), and although this was mostly consistent with a benefit, it was based on only two studies.

**Conclusion:**

Our data support that moderate to high levels of PA are associated with a 21% lower risk of all-cause and 24% cardiovascular disease mortality in adults with obesity. Although data from the only two published studies seem to indicate a protective effect of PA on cancer risk, the estimates are not statistically significant.

**Systematic review registration:**

PROSPERO CRD42022309346.

**Supplementary Information:**

The online version contains supplementary material available at 10.1186/s12889-024-19383-z.

## Introduction

Obesity has become a global epidemic, reaching alarming proportions in recent decades, and poses a major public health challenge [1]. According to the World Health Organization (WHO) in 2016, more than 1.9 billion adults aged 18 years and older were overweight, and of those, over 650 million adults were obese; if this trend continues, a majority of the world’s adult population will be either overweight or obese by 2030 [2].

Obesity is characterized by an excessive accumulation of body fat, which is associated with several adverse health outcomes, including type 2 diabetes, cardiovascular disease (CVD), heart failure, and some cancers, leading to an increased all-cause mortality risk [3].

Conversely, consistent evidence from observational studies shows that physical activity (PA) reduces the risk of CVD, cancer, and all-cause mortality in adults [4, 5]. In addition, several meta-analyses synthesizing the evidence from population-based adult follow-up studies in which PA was device-measured have shown that PA, regardless of its intensity (light, moderate, or vigorous), is associated with a lower risk of all-cause mortality [6, 7]. Similarly, a study pooling data from eight cohorts reported a dose–response relationship between total and intensity-specific PA and mortality in individuals with normal weight (according to their body mass index (BMI) 18.5–24.9 kg/m^2^) and with overweight (BMI 25–29.99 kg/m^2^), but in individuals with obesity (BMI ≥ 30.9 kg/m^2^) this relationship was observed only for total PA [8]. Despite all this evidence, more than a quarter of the world's population does not meet the recommended minimum levels of PA [9]. Additionally, high-income countries have a greater prevalence of not meeting these recommended levels [10].

Both the high prevalence of obesity and the low levels of PA are currently major public health challenges worldwide because of the burden they place on both economies and healthcare systems [11]. Although it has been suggested that PA may play a key role in the prevention and management of obesity [12], previous evidence indicates that the response to exercise in terms of adiposity is individually variable, and the relationship is highly complex [13] However, the health benefits of PA go beyond whether weight loss occurs and include, in addition to its effects at the cellular level (changes in telomere length, greater efficiency in mitochondrial respiration and improvements in oxidative stress homeostasis), [14, 15] improvements in the lipid profile, increased insulin sensitivity and reduced liver fat [16]. All of these metabolic improvements, together with improvements in physical fitness [16], lead to reductions in mortality and healthcare costs far beyond those associated with weight loss [17]. Finally, although only a few studies have examined the benefits of PA in individuals with obesity from a gender perspective, [18] the benefits of PA on mortality in people with obesity have been described in both women [19] and men [20].

However, although a large number of systematic reviews and meta-analyses have estimated the effect of PA on mortality in population-based adults [6, 8, 21, 22], as well as in individuals with various diseases [23–29], a synthesis of the available evidence on the effect of PA on mortality risk in people with obesity is lacking. Therefore, our systematic review and meta-analysis aimed to estimate the effect of PA levels on mortality (all-cause, cardiovascular, and cancer) in adults with obesity.

## Methods

The Cochrane Collaboration Handbook [30], the Preferred Reporting Items for Systematic Reviews and Meta-Analyses [31], and the MOOSE guidelines [32] guided the present study. Previously, the protocol was registered in the PROSPERO database (CRD42022309346).

### Deviation from the protocol

This review deviates from the protocol on the following issues: we did not include studies on cardiovascular or cancer incidence because of insufficient data; a change in authorship was made after submitting the PROSPERO protocol (March 2022).

### Data sources and searches

Two reviewers (RF-R and SNA-A) independently searched the MEDLINE (via PubMed), Embase (via Scopus), Web of Science (WoS) and SPORTDiscus (via EBSCOhost) databases, from inception to October 5th 2023, to identify prospective cohort studies aimed at determining the association between moderate to high PA and mortality (all-cause mortality, cardiovascular mortality, and cancer mortality) in adults with obesity (body mass index [BMI]: ≥ 30 kg/m^2^]. The search syntax included the following terms: obese [MeSH Terms] or obesity [All fields] or "excess body fat"[All fields] and Exercise[MeSH Terms] or "physical activity"[All fields] and mortality[MeSH Terms] or "all-cause mortality" or "cardiovascular mortality" or death[All fields] and cohort[MeSH Terms] or “observational study” [All fields]. No language restrictions were applied. The Mendeley desktop find and merge duplicated tool was used to search for duplicates and a third reviewer peer-reviewed the search process (VMV). Further details of the search strategy employed for each database are available in Table S1.

### Study selection

The PI(E)COs strategy was followed to determine the inclusion criteria. Therefore, our inclusion criteria were as follows: i) type of studies: prospective cohort studies; ii) participants: adults aged ≥ 18 years with a body mass index (BMI) ≥ 30 kg/m^2^; iii) exposure: PA, defined as “any bodily movement produced by skeletal muscles that requires energy expenditure”; [33] thus, any type of PA determined through questionnaires or device-measurements was considered, and we used the PA categories reported from primary studies iv) comparator or reference: insufficiently active category based on primary studies, including those defined as sedentary (any waking behaviour characterized by an energy expenditure of 1.5 METS or lower) but also, insufficiently active categories determined by quartiles, quintiles or similar; [33] v) outcomes: all-cause mortality, cardiovascular mortality or cancer mortality. When data were duplicated in publications, the most recent studies with the longest follow-up or the largest sample size were considered.

We excluded prospective studies when they did not report the specific number of cases and total sample across participants with a BMI ≥ 30 kg/m^2^ for the PA categories of interest (insufficiently active vs. active [moderate to high PA]). Case reports and letters were excluded. The list of studies for full-text reading and their specific reasons for exclusion are available in Table S2.

### Data extraction

Two authors (RF-R and AT-C) independently extracted the following information from each included study: 1) first author name and publication year, 2) country and cohort name, 3) follow-up length (years), 4) sample characteristics: sample size, percentage of females, mean/range for age, mean/range for BMI, reported comorbidities (hypertension, CVD, diabetes, etc.), percentage of smoking status and alcohol consumption, 5) PA categories of each study, 6) mortality outcome (all-cause, cardiovascular, and cancer), and 7) adjustment variables for the analyses. Sample characteristics were reported for participants with obesity, when possible; otherwise, data are presented for all cohort participants. Regarding outcomes, we considered all-cause, cardiovascular, and cancer mortality as the main outcomes. A third researcher (ER-G) independently appraised the accuracy of the extracted information.

### Quality assessment

Each study was evaluated independently by two authors (MGM and RFR) in each of the domains of bias contained in the Risk of Bias in Non-randomized Studies-of Exposure ROBINS-E tool (for follow-up studies) [34]. The validity of the studies was judged for seven potential bias domains: i) confounding, ii) measurement of the exposure, iii) selection of participants into the study (or into the analysis), iv) post-exposure interventions, v) missing data, vi) measurement of the outcome, and vii) selection of the reported result. Each bias domain was assessed using a series of signalling questions. The risk of bias was rated as low, with some concerns, as high or very high.

### GRADE report

The “Grades of Recommendations, Assessment, Development, and Evaluation” (GRADE) tool was used to determine the certainty of the evidence of the present systematic review [35]. Each outcome was rated as having high-, moderate, low-, or very low-quality evidence based on the design of the studies, risk of bias, inconsistency, indirect evidence, imprecision, and publication bias. Accordingly, the score was downgraded one when < 75% of the analyzed studies were at low risk of bias, as well as when inconsistency (I2 > 50%), indirect evidence, imprecision (wide confidence intervals) and publication bias were reported.

### Data synthesis

When at least two studies reported the effect estimate for the same outcome, a meta-analysis was conducted considering the most adjusted effect estimates and their 95% confidence intervals (CIs). The reported hazard ratios (HRs) were considered equivalent to relative risks (RRs); moreover, because most studies provided HRs the analyses were performed using this effect estimate. Three studies [36–38]. reported RRs, therefore, a sensitivity analysis was conducted by removing the estimates to determine whether the different types of effect estimates could modify the results. For the meta-analysis of the influence of active (moderate to high PA), their effect estimates were compared to insufficiently active categories (reference categories from included studies); then, we combined the effect estimates using the random-effects model of DerSimonian and Laird [39]. Moreover, in cases of included studies that reported two or more exposed groups according to moderate to high PA values, we used a fixed-effects model with the inverse variance method to obtain the pooled effect estimate [40]. Likewise, when the included studies provided data stratified by BMI status (30 to 34.99 vs. ≥ 35 kg/m^2^) [36], or sex (male vs. female), we pooled the most adjusted effect estimates. Statistical heterogeneity between studies was examined using the I^2^ statistic; thus, I^2^ values of 0%—40% were considered ‘not important’ heterogeneity, 30% to 60% represented ‘moderate’ heterogeneity, 50% to 90% represented ‘substantial’ heterogeneity, and 75% to 100% represented ‘considerable’ heterogeneity. We considered their corresponding *p*-values and 95% CIs [41].

Meta-regression models considering total sample size, sample size for insufficiently active and active (moderate to high PA) categories, and length of follow-up to determine their influence on the effect estimates were conducted. When at least three studies reported data for the same outcome a sensitivity analysis was performed excluding the effect estimates from the included studies one by one to evaluate the influence of each study on the pooled effect estimate.

All the statistical analyses were performed using StataSE v. 15 (StataCorp, College Station, TX, USA).

## Results

### Literature search

**Fig. 1:**
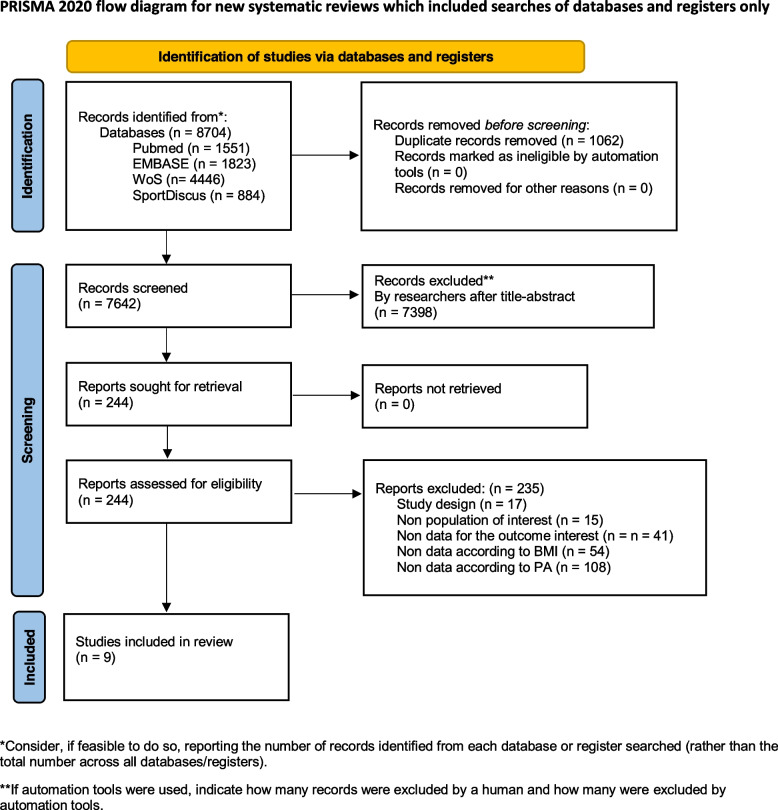
Flow diagram of studies through the review (PRISMA 2020)

A total of 8704 studies were identified through systematic searches, of which 1062 duplicated records were removed. Finally, after the full-text review of the 244 studies assessed for eligibility, nine prospective cohort studies were included in the systematic review and meta-analysis with data on three outcomes: all-cause mortality (*n* = 8) [36–38, 42–46], cardiovascular mortality (*n* = 2) [36, 46] and cancer mortality (*n* = 2) (Fig. 1) [36, 47]. The reasons for exclusion of the studies excluded after the full-text review are available in the supplementary material (Table S2).

### Study characteristics

The main characteristics of the studies included are available in Table 1. All studies were published between 2002 and 2021. The country of origin of the cohorts varied; three studies were conducted in the USA [37, 46, 47] one study was conducted with multicountry data from the EPIC cohort [44], and one study was from the UK [36] Spain, [42] Korea [45], the Netherlands [43], and Puerto Rico [38]. The total number of participants included in the studies ranged from 781 to 64,908. A total of 199,425 participants were considered, of whom 59,873 were in the insufficiently active category and 84,328 were in the active, moderate to high PA, category. The range of follow-up was between 6 and 13 years (mean: 9.5 y). Most studies were conducted with both male and female participants, except for one study that included only males [38], and one that included only females [47]. The age range for the included participants was between 35 and 85 years, and all participants were categorized as having obesity according to their BMI (≥ 30 kg/m^2^). Most studies measured BMI under standardized conditions, only Min et al. [45] and Patel et al. [37] did not report how this variable was measured. There was some percentage of participants among the prospective cohorts who presented comorbidities at baseline, such as CVD (11.3%) [43], cancer (5.2% to 9.1%) [43, 47] hypertension (35.4% to 73.7%) [36, 38, 46] diabetes (4.5% to 21.7%) [36, 46] or high blood cholesterol (15.9%) [38]. Generally, smoking habits were also reported, with prevalence values between 6.9% and 38.8%.

**Table 1:** Characteristics of the included studies through the review

Reference	Country (cohort)	Follow-up (y)	n (% female)	Age (years), mean/range	BMI (kg/m^2^), mean/range	Comorbidities	Smoke; alcohol	Exposure; PA categories	Outcome
Balboa-Castillo T et al., [42]	Spain (ENRICA)	8	955 (NR) Sedentary: 204 Active: 751	≥ 60 (all cohort)	≥ 30	NR	NR	Continually sedentary/Decreased leisure time PA/ Increased LTPA/continually active	ACM
Crespo et al., [38]	Puerto Rico (Puerto Rico Heart Health Program)	12	781 (0) Sedentary: 341 Active: 440	35 to 79 (all cohort)	≥ 30	Hypertension 35.4%, high blood cholesterol 15.9% (All cohort)	Non-smokers 34.2%; previous smokers 22.1%; smokers (1 to > 20): 29.3% (All cohort)	Quartiles after Framingham PA index* (Q1 = reference, sedentary group)	ACM
de Boer et al., [43]	Netherlands (LifeLines)	7.7	15,254 (61.6) Sedentary: 4058 Active: 11,196	48.0	32.4 (median)	CVD baseline 11.3%, Cancer at baseline 5.2%	Current smokers: 17.7%, former smokers: 38.8%; Alcohol: no/little (< 1glass/week) 25.8%, heavy (> = 5days/week) 8.8%	SQUASH questionnaire (frequency and duration of participation in several types of PA: cycling, gardening, odd jobs, walking)	ACM
Ekelund et al., [44]	Multicountry. (EPIC)	12.4	52,903 (64.2) Sedentary: 32,355 Active: 20,548	Males: 52.6 ± 9.6; Females: 51.2 ± 10.3 (all cohort)	≥ 30	NR	Never smokers: 44.0%, current smokers: 26.0%	Inactive (36 kJ/kg daily); moderately inactive (41 kJ/kg daily); moderately active (46 kJ/kg daily), and active (51 kJ/kg daily)	ACM
Min C et al., [45]	Korea (NHIS-NSC)	6	9400 (44.1 all cohort) Sedentary: 5019 Active: 4381	45 to 85 (all cohort)	≥ 30	Charlson comorbidity index 0.58 (1.30)	NR	Insufficient PA or PA considered as: Moderate PA ≥ 5 d/week for ≥ 30 min or vigorous PA ≥ 3 d/week for ≥ 20 min	ACM
Patel AV et al., [37]	USA (CPS -II)	13	18,358 (58.9)	Males: 63.6 ± 6.0; Females: 61.9 ± 6.5 (all cohort)	≥ 30	NR	NR	METs categories: 17.5 to < 24; 24.5 to < 31.5;31.5 to < 42;42 to < 52.5; 52.5 to < 63; > = 63 METs/hour/week	ACM
Sanchez-Lastra M et al., [36]	UK (UK Biobank Resource)	8.9	64,908 (48.8) Sedentary: 17,896 Active: 47,012	37 to 82 (all cohort; mean: 56.0)	Class I: 30 to 34.99, Class II: ≥ 35	Diabetes: 4.55% / Hypertension: 51.97% (all cohort)	Never smokers: 53.1%	Three categories of PA after quintiles classification by METs/minutes/week: Low physical activity (Q1), medium physical activity (Q2 and Q3), and high physical activity (Q4 and Q5)	ACM, CVM, CM
Wang A et al., [47]	USA (WHI)	6	33,568 (100%)	50 to 79 (all cohort, mean: 63.2)	≥ 30	History of cancer: 9.13%; asthma: 7.79; emphysema or chronic bronchitis: 3.62%	Never smokers 52.0%, former smokers 41.0%, current smokers 6.93% / Alcohol: non-drinker 10.8%, past drinker 18.3%, drinker 71.0%	Four categories by METs/min/week: 0 to < 100 (inactive), 100 to < 500 (low), 500 to < 1200 (medium), > 1200 (high)	CM
Willey JZ et al., [46]	USA (NOMAS)	11.8	3298 (62.8 all cohort)	69.2 ± 10.3 (all cohort)	≥ 30	Hypertension: 73.7%; Diabetes: 21.7% (all cohort)	Never smokers 46.8%, former smokers 36.1%, current smokers 17% / Alcohol: never or heavy: 67.1%, mild-moderate: 32.7% (all cohort)	Three categories: insufficiently active (reference), intermediate level of METs-score, and the highest level of METs-score	ACM, CVM

The data are reported for people with obesity if possible, and if not, for all cohort populations. *HR* hazard ratio, *RR* risk ratio, *ACM* all-cause mortality, *CVM* cardiovascular mortality, *CM* cancer mortality, *CVD* cardiovascular disease, *USA* United States of America, *UK* United Kingdom, *PA* physical activity, *SQUASH* short questionnaire to assess health-enhancing physical activity, *NR* not reported

^*^Framingham PA index assesses occupational, leisure time and other physical activities, measured as usual activity (hours spent at various activities) over the course of a 24-h day (interviewer-administered), converted to an index of usual daily energy expenditure, categories using estimated oxygen consumption per hour for each activity (METs). Sedentary = 1MET, slight = 1.1 to 2.3 MET, slightly moderate to moderate = 2.4 to 4.9MET, strenuous = 5 + MET

### Exposure

We dichotomized participants in the included studies according to their usual levels of PA into insufficiently active (reference category) and active (moderate to high PA) (Table S3). Those who were insufficiently active were: sedentary [42], in the first quartile Q1 of the after Framingham PA Index [38], not included in the active category [43, 45], inactive at 36 kJ/kg daily [44], with < 17.5 MET/h/week [37], in the first quintile of the IPAQ-SF [36], with 0 to < 100 METs/min/week [47] and not active [46].

The following were considered active: leisure time or continuous PA [42], quartiles Q3-Q4 according to the Framingham PA Index [38], cycling, walking and sports participation, [43] moderately active (46 kJ/kg daily) and active (51 kJ/kg daily), [44] moderate PA ≥ 5 d/week for ≥ 30 min or vigorous PA ≥ 3 d/week for ≥ 20 min [45], from 17.5 to > 63 MET/h/week [37] Q2-Q5 [36], or medium and most active, from 500 to > 1200 MET-min/week [47] and Q3-Q4 [46].

### Outcomes and adjustments

Most studies assessed all-cause mortality [36–38, 42–46]. In addition, two of them assessed cardiovascular mortality [36, 46], and there were two studies [36, 47] in which cancer mortality was assessed, with the main outcome being one [47]. Among them the adjustment variables were diverse, but most were related to age, sex, socioeconomic and health status, and lifestyle. Further details about the specific adjustments made in each included study are available in Table 2.

**Table 2:** Quality score and adjustment variables for the analysis of the included studies

Reference	NIH score	Adjustment
Balboa-Castillo T et al., [42]	Good	HRs for ACM adjusted by age, sex, educational level, smoking, alcohol, chronic diseases (coronary disease, stroke, cancer, chronic obstructive pulmonary disease, diabetes, hip fracture), physical activity, cognitive performance, limitations in daily living activities, mobility, or agility
Crespo et al., [38]	Good	RRs for ACM adjusted by age, smoking, education, urban residence, hypertension, and high blood cholesterol
de Boer et al., [43]	Fair	HRs for ACM adjusted by age, sex, education, net household income, alcohol consumption, smoking status, diet quality, health status (depression, burnout, diagnosed CVD, cancer), amount of leisure-time and subjective well-being
Ekelund et al., [44]	Fair	HRs for ACM, adjusted by sex, education, smoking, and alcohol
Min C et al., [45]	Good	HR for ACM adjusted by age, sex, income, region of residence, smoking, alcohol consumption, and Charlson comorbidity index
Patel AV et al., [37]	Fair	RRs for ACM adjusted by age, race, marital status, smoking, alcohol, total caloric intake, comorbidities score
Sánchez-Lastra M et al., [36]	Good	HRs for ACM in never-smokers, HRs of ACM by sex, RRs for CVM, and CM, adjusted by age, sex, ethnicity, education, marital status, employment, Townsend deprivation index, diet pattern, salt intake, alcohol intake, smoking status, screen time, asthma, depression, hormone replace therapy (women only), diabetes, hypertension, and statins medication
Wang A et al., [47]	Good	HRs for lung cancer mortality adjusted by age, race/ethnicity, body mass index (BMI), family history of cancer, personal history of cancer (except lung), smoking, education, history of asthma, history of emphysema or chronic bronchitis, alcohol intake, vitamin D use, hormone therapy use, oral contraceptive use, NSAID use, hormone therapy trial arm, DM trial, CVD trial arm, OS/CT, hysterectomy status, servings of fruit, vegetables, and red meat
Willey JZ et al., [46]	Fair	HRs for ACM adjusted by age, race-ethnicity, high school education, health insurance, moderate alcohol use, tobacco use, hypertension, diabetes, LDL-cholesterol, HDL-cholesterol, and any heart disease

*HR* hazard ratio, *RR* risk ratio, *ACM* all-cause mortality, *CVM* cardiovascular mortality, *CM* cancer mortality, *CVD* cardiovascular disease, *NSAID* nonsteroidal anti-inflammatory drugs, *DM* dietary modification, *OS/CT* time from the date of entry into study to onset of lung cancer (incidence) or to death from lung cancer (mortality)

### Risk of bias and certainty of evidence

The risk of bias assessment results obtained using the ROBINS-E tool [34], are shown in Supplementary Table S4. The studies selected for the review showed variability in the different quality assessment domains. Four studies had a low risk of bias in all domains analyzed [37, 42, 45, 47]. Most studies were judged with some concerns due to incomplete adjustment for all confounders, statistical analysis, missing data and reporting of results.

The overall certainty of the evidence was determined only for all-cause mortality and was set as ‘very low certainty’ (*n* = 8) (Supplementary Table S5). For cardiovascular (*n* = 2) and cancer (*n* = 2) mortality outcomes, we did not determine the certainty of evidence due to the scarcity of included studies.

### All-cause mortality

**Fig. 2:**
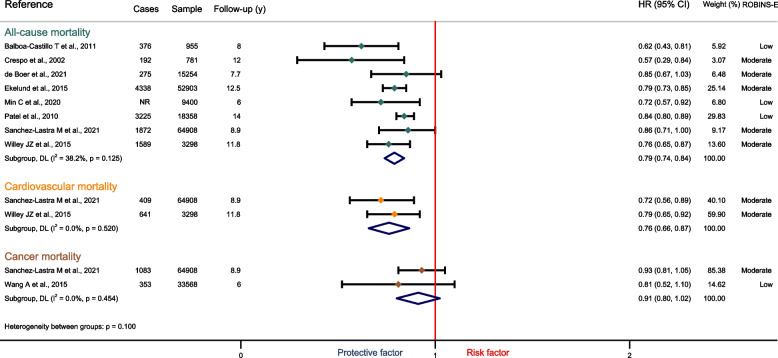
Forest plot for Hazard Ratios and Risk Ratios for the effect of physical activity on mortality in adults with obesity

Eight studies [36–38, 42–46], with a total of 165,857 participants, among which 59,873 were in insufficiently active, and 84,328 in moderate to high PA categories were included. Moderate to high PA was associated with a 21% lower risk of all-cause mortality (HR: 0.79, 95%CI: 0.74 to 0.84; I^2^ = 38.2%, *p* = 0.13) in people with obesity (Fig. 2). Similar results were found in adults with overweight (HR: 0.79, 95%CI: 0.72 to 0.86; I^2^ = 85,9%, *p* = 0.000; Fig. S1).

### Cardiovascular disease mortality

Two studies [36, 46] with a total of 65,809 participants, among which 17,896 were in insufficiently active, and 47,012 moderate to high PA categories. Moderate to high levels of PA were associated with a 24% lower risk of cardiovascular mortality (HR: 0.76, 95%CI: 0.66 to 0.87; I^2^ = 0.0%, *p* = 0.52) in people with obesity (Fig. 2). Only one study reported results in adults with overweight [36].

### Cancer mortality

Two studies [36, 47] with a total of 98,476 participants, among which 17,896 were in insufficiently active, and 47,012 moderate to high PA categories. Moderate to high PA was not significantly associated with the risk of cancer mortality (HR: 0.91, 95%CI: 0.80 to 1.02; I^2^ = 0.0%, *p* = 0.45) in people with obesity (Fig. 2). Similar results were found in adults with overweight (HR: 0.85, 95%CI: 0.66 to 1.04; I^2^ = 61,9%, *p* = 0.105; Fig. S1).

### Sensitivity analyses and meta-regression models

The sensitivity analysis excluding studies one by one showed similar significant results for all-cause mortality (Supplementary Table S6**)**. Moreover, when meta-regression models were conducted according to the length of follow-up (y), total sample size and number of participants in the active and insufficiently active groups, no significant differences were found. Finally, publication bias was significant for all-cause mortality (coefficient: 0.86; *p* = 0.08) (Supplementary Table S7).

## Discussion

Our study is the first to synthesize the available evidence about the association between PA and mortality risk in individuals with obesity. Our analyses support that moderate to high levels of PA are associated with a 21% lower risk of all-cause mortality and with a 24% lower risk of cardiovascular mortality in people with obesity. However, our analyses did not suggest that moderate to high levels of PA reduced the risk of cancer mortality. Although the estimates of the association between PA and the risk of all-cause mortality appear to be clear, at least when different grades of obesity are combined, both cancer and cardiovascular associations are based on the results of only two follow-up studies, and more studies are needed to draw robust conclusions about these associations.

Obesity is associated with a significantly greater risk of all-cause mortality than a normal weight [48, 49]. Several epidemiological studies have provided evidence suggesting that PA reduces all-cause mortality in people with obesity. First, a systematic review of follow-up studies reported that, in most studies, the relationship between obesity and mortality disappeared after adjustment for PA [50]. In addition, other studies reported that PA significantly reduced the risk of death associated with high BMI [51]. This finding is consistent with the results of a recent study that jointly analyzed the time course of excess adiposity and PA in Taiwan's MJ Cohort, which revealed that increases in PA reduced the risk of all-cause mortality, whereas decreases in adiposity mitigated but did not offset the risk of all-cause mortality [52]. Our analyses of data from eight cohort studies involving 165,857 participants are consistent with these previous findings, suggesting that moderate to high levels of PA were associated with a 21% reduction in the risk of all-cause mortality. In addition, as evidence of consistency in our estimates, all studies included in this meta-analysis showed a reduction in mortality, although this difference was significant in six of the eight studies. Chronic physiological adaptations to physical activity, such as improved cardiac performance, increased oxygen uptake, improved insulin sensitivity or decreased blood pressure, among others, could explain the reduction in mortality risk, resulting in improved cardiovascular function, improved metabolic health or reduced chronic inflammation [11, 53].

Given that CVD and cancer are the two leading causes of death worldwide, and that both are associated with both obesity and PA [51], it is not surprising that estimates of the association between these two major disease groups and PA in individuals with obesity are consistent with previous estimates of all-cause mortality risk. Regarding cardiovascular mortality, the association of obesity with most cardiovascular risk factors and the benefits of increased PA in improving these cardiovascular risk factors and the risk of death from CVD have been extensively studied in both healthy adults and populations with underlying cardiovascular pathology. However, the two studies included in our review are the only available estimates of the effect of moderate to high levels of PA in people with obesity, showing significant reductions of 28% [36] and 21% [46], respectively. Although the weakness of our pooled estimates, which were obtained from only two studies is obvious, recent publications on increasing PA during follow-up seem to support the beneficial effect of exercise on CVD mortality [52].

As in the case of the association between PA and all-cause mortality risk, the data from the studies included in our analyses show that PA may reduce the risk of death from cancer, but more modestly, the risk ranges from 7% [36], for all cancers to 19% for lung cancer in women [47], which is not statistically significant. The lack of statistical power of these estimates may be because cancer is not a single clinical entity but rather a family of pathological entities in which the role of PA as a protective factor may be heterogenous and in which the importance of other risk factors, such as smoking, may vary considerably from one type of cancer to another. Cancer is therefore a group of pathologies in which the preventable fraction of the mortality risk is highly variable, once risk factors or potential confounding variables are taken into account. Therefore, in addition to studies analyzing the association between PA and cancer mortality risk, studies analyzing this risk in specific types of cancer, such as breast, colorectal or lung cancer, are needed.

Although high levels of PA may help maintain a healthy body weight [36], it is also plausible that PA has health and longevity benefits independent of maintaining a healthy body weight. These effects may include favourable glycemic control [54], and lower blood pressure [36], which are considered important mediators of adiposity-related morbidity and mortality [36]. It is therefore possible that mortality risk is a function of the combined effect of PA and BMI. Current evidence suggests that the increased risk of premature death in people with high adiposity is reduced, but not eliminated, by PA [36].

### Limitations

This systematic review and meta-analysis of prospective cohort studies has several limitations that need to be understood to fully appreciate the extent of the estimates presented. First, we included only subjects with obesity (BMI ≥ 30 kg/m^2^) and, therefore did not include individuals with overweight, because whether, depending on age, overweight is a risk factor for mortality is a controversial issue [55]. Despite this, we have analyzed the estimates for people with overweight (after peer review) and included these results in the supplementary material. We used the 30kg/m^2^ cut-off for adults suggested by the World Health Organization as a consensus [56]. However, for older people, some authors have suggested lower cut-off points, such as 27 kg/m^2^ [57]. Therefore, given the age range of the participants included in the study, some obese older people may not have been included in the research. We also included people with type I and severe obesity, but the risk may be different in each category [48]. Second, in most studies the quantification of PA is based on data from self-reported PA questionnaires, which adds heterogeneity to the measurement of PA exposure and prevents us from knowing how much PA is needed to reduce mortality. Third, studies do not consider trajectories (changes) in both PA levels and weight status during follow-up, and these changes may provide valuable information to confirm the extent to which PA increases or decreases outcomes. Fourth, the variability in the control of covariates in the studies or the fact that the association between PA and obesity with risk of all-cause mortality may differ by age group, threatens the validity of our estimates, which cannot be controlled with the data provided by the studies. The wide variability of comorbidities reported by the studies concerning the participants included in the studies has meant that they could not be taken into account in the analyses. Finally, in observational studies, the possibility of reverse causality cannot be ruled out. There is therefore a need for long-term randomized trials in individuals with obesity to assess the effectiveness of physical activity on mortality; given the difficulty and cost of such studies, pragmatic trials using large databases (i.e. the UK Biobank) could elucidate this association without the threat of reverse causality biases.

## Conclusions

Our systematic review and meta-analysis suggest that moderate to high levels of PA can substantially reduce the risk of all-cause and CVD mortality. In the case of cancer, although the few available data also seem to indicate a beneficial effect, the available evidence does not allow us to conclude the potential benefits of PA in reducing mortality. The data from this study confirm that PA could play a major role in preventing the risk of death in people with obesity in this new era of effective (and expensive) pharmacological treatment, but it is also one of the most difficult tools to work with, as many people find it difficult to increase PA levels, and this is currently a challenge. In addition, our estimates are not sufficiently robust, so further studies that include more PA device measures for the exposure, such as accelerometers or 24-h activity trackers, and measures of obesity that include more precise assessments of adiposity in addition to BMI are needed.

### Supplementary Information

Supplementary Material 1.

## Data Availability

Data sharing: Data described in the manuscript, code book, and analytic code will be made available upon request pending to the corresponding author.

## Abbreviations

BMI: Body mass index
CVD: Cardiovascular disease
HR: Hazard ratio
PA: Physical activity
RR: Relative risk
