# Supplementary material for: Physical activity is associated with lower mortality in adults with obesity: a systematic review with meta-analysis
Source: BMC Public Health. 2024 Jul 12;24:1867. doi: 10.1186/s12889-024-19383-z (PMC11245862; doi:10.1186/s12889-024-19383-z)
Supplement: Supplementary file 1 — Supplementary Material 1. [file 12889_2024_19383_MOESM1_ESM.docx]

**SUPPLEMENTARY MATERIAL**

**Physical activity is associated with lower mortality in adults with obesity: a systematic review and meta-analysis of cohort studies.**

**Running title:** Physical activity and mortality in adults with obesity.

Vicente Martínez-Vizcaíno,^1,2^ Rubén Fernández-Rodríguez,^1*^ Sara Reina-Gutiérrez,^1^ Eva Rodríguez-Gutiérrez,^1^ Miriam Garrido-Miguel,^1,3^ Sergio Núñez de Arenas-Arroyo,^1^ Ana Torres-Costoso.^1,4^

1. University of Castilla-La Mancha, Health and Social Research Center, Cuenca, Spain.
2. University Autonomous of Chile, Faculty of Health Sciences, Talca, Chile.
3. University of Castilla-La Mancha, Faculty of physiotherapy and nursing, Toledo, Spain.
4. University of Castilla-La Mancha, Faculty of nursing, Albacete, Spain.

**Corresponding author**: Rubén Fernández-Rodríguez.

University of Castilla-La Mancha, Health and Social Research Center, Santa Teresa Jornet s/n, 16071 Cuenca, Spain

E-mail: ruben.fernandez@uclm.es

**Key words**: Mortality, Cancer, Physical activity, Obesity.

**Acknowledgements**: None.

**Potential conflicts of interest**: The authors declare no conflicts of interest.

### **Index**:

| ID | Subheading | Page number(s) |
| --- | --- | --- |
| Table S1 | Search strategy for each database | 3-4 |
| Table S2 | Studies excluded after full text reading with reasons | 5-15 |
| TABLE S3 | Definitions of physical activity categories (Insufficiently active and active) from original studies | 16 |
| table S4 | Quality assessment with ROBINS-E tool | 17 |
| TABLE S5 | GRADE summary of findings | 18 |
| TABLE S6 | Sensitivity analysis | 19 |
| TABLE S7 | Meta-regression models and publication bias | 20 |

**Table S1.** Search strategy for each database. Date: 28^th^, June 2024.

| **MEDLINE (via Pubmed)** |
| --- |
| \| **Search number** \| **Query** \| **Results** \| \| --- \| --- \| --- \| \| 5 \| (#1) AND (#2) AND (#3) AND (#4) \| **1,551** \| \| 4 \| "observational study"[Publication Type] OR "observational studies as topic"[MeSH Terms] OR "observational study"[All Fields] OR ("cohort studies"[MeSH Terms] OR ("cohort"[All Fields] AND "studies"[All Fields]) OR "cohort studies"[All Fields] OR "cohort"[All Fields] OR "cohort s"[All Fields] OR "cohorte"[All Fields] OR "cohorts"[All Fields] OR "follow-up" [All Fields]) \| 3,724,859 \| \| 3 \| "mortality"[MeSH Terms] OR "mortality"[All Fields] OR "mortalities"[All Fields] OR "mortality"[MeSH Subheading] OR "all-cause mortality"[All Fields] OR "cardiovascular mortality"[All Fields] OR ("death"[MeSH Terms] OR "death"[All Fields] OR "deaths"[All Fields]) \| 2,493,323 \| \| 2 \| "exercise"[MeSH Major Topic] OR "physical activity"[All Fields] OR ("exercise"[MeSH Terms] OR "exercise"[All Fields] OR "exercises"[All Fields] OR "exercise therapy"[MeSH Terms] OR ("exercise"[All Fields] AND "therapy"[All Fields]) OR "exercise therapy"[All Fields] OR "exercise s"[All Fields] OR "exercised"[All Fields] OR "exerciser"[All Fields] OR "exercisers"[All Fields] OR "exercising"[All Fields]) \| 677,932 \| \| 1 \| "obeses"[All Fields] OR "obesity"[MeSH Terms] OR "obesity"[All Fields] OR "obese"[All Fields] OR "obesities"[All Fields] OR "obesity s"[All Fields] OR ("obeses"[All Fields] OR "obesity"[MeSH Terms] OR "obesity"[All Fields] OR "obese"[All Fields] OR "obesities"[All Fields] OR "obesity s"[All Fields]) OR "excess body fat"[All Fields] \| 481,983 \| |
| **EMBASE (via Scopus)** |
| \| **#** \| **Search Query** \| **Results** \| \| --- \| --- \| --- \| \| 5 \| #1 AND #2 AND #3 AND #4 \| **1,823** \| \| 4 \| TITLE-ABS-KEY ( ( cohort ) OR ( "observational study" ) ) \| 1,600,016 \| \| 3 \| TITLE-ABS-KEY ( ( mortality OR "all-cause mortality" OR "cardiovascular mortality" OR death ) ) \| 3,196,048 \| \| 2 \| TITLE-ABS-KEY ( ( exercise OR "physical activity" OR exercise ) ) \| 1,012,267 \| \| 1 \| TITLE-ABS-KEY (( obese OR obesity OR "excess body fat")) \| 667,217 \| |
| **Web Of Science (All databases)** |
| \| **#** \| **Search Query** \| **Results** \| \| --- \| --- \| --- \| \| 5 \| #1 AND #2 AND #3 AND #4 \| **4446** \| \| 4 \| TS=(cohort OR "observational study" ) \| 1,668,427 \| \| 3 \| TS=(mortality or "all-cause mortality" or "cardiovascular mortality" or death) \| 4,197,333 \| \| 2 \| TS=(Exercise or "physical activity") \| 1,369,254 \| \| 1 \| TS=(obese or obesity or "excess body fat") \| 1,004,526 \| |
| **SPORTdiscuss(via EBSCOhost)** |
| \| **Search number** \| **Query** \| **Results** \| \| --- \| --- \| --- \| \| 5 \| (#1) AND (#2) AND (#3) AND (#4) \| **884** \| \| 4 \| AB ( cohort   OR  "observational study" ) \| 29,698 \| \| 3 \| TX (mortality or "all-cause mortality" or "cardiovascular mortality" or death) \| 139,293 \| \| 2 \| TX( Exercise or "physical activity") \| 465,095 \| \| 1 \| TX (obese or obesity or "excess body fat") \| 82,460 \| |

Table S2. Studies excluded after full text reading with the reason for exclusion.

| **ID** | **Reference** | **Eligibility** | **Reason for exclusion** |
| --- | --- | --- | --- |
|  | Leitzmann MF, Park Y, Blair A, et al. Physical activity recommendations and decreased risk of mortality. Arch Intern Med. 2007;167(22):2453-2460. doi:10.1001/archinte.167.22.2453 | Excluded | Non-data of interest according to BMI |
|  | Li X, Wu C, Lu J, et al. Cardiovascular risk factors in China: a nationwide population-based cohort study. Lancet Public Heal. 2020;5(12):e672-e681. doi:10.1016/S2468-2667(20)30191-2 | Excluded | Non-data for outcome of interest |
|  | Li X, Chen K, Hua W, et al. Association of the Obesity Paradox With Objective Physical Activity in Patients at High Risk of Sudden Cardiac Death. J Clin Endocrinol Metab. 2020;105(12). doi:10.1210/clinem/dgaa659 | Excluded | Non-data of interest according to BMI |
|  | Liakopoulos V, Franzén S, Svensson A-M, et al. Changes in risk factors and their contribution to reduction of mortality risk following gastric bypass surgery among obese individuals with type 2 diabetes: A nationwide, matched, observational cohort study. BMJ Open Diabetes Res Care. 2017;5(1). doi:10.1136/bmjdrc-2016-000386 | Excluded | Non-data of interest according to PA |
|  | Lidin M, Hellénius M-L, Rydell-Karlsson M, Ekblom-Bak E. Long-term effects on cardiovascular risk of a structured multidisciplinary lifestyle program in clinical practice. BMC Cardiovasc Disord. 2018;18(1). doi:10.1186/s12872-018-0792-6 | Excluded | Non-data for outcome of interest |
|  | Lin W-Y, Tsai S-L, Albu JB, et al. Body mass index and all-cause mortality in a large Chinese cohort. C Can Med Assoc J = J l’Association medicale Can. 2011;183(6):E329-36. doi:10.1503/cmaj.101303 | Excluded | Non-data of interest according to PA |
|  | Lin Y, Kikuchi S, Tamakoshi A, et al. Obesity, physical activity and the risk of pancreatic cancer in a large Japanese cohort. Int J cancer. 2007;120(12):2665-2671. doi:10.1002/ijc.22614 | Excluded | Non-data of interest according to PA |
|  | Lin Y-K, Wang C-C, Yen Y-F, et al. Association of body mass index with all-cause mortality in the elderly population of Taiwan: A prospective cohort study. Nutr Metab Cardiovasc Dis. 2021;31(1):110-118. doi:10.1016/j.numecd.2020.08.014 | Excluded | Non-data of interest according to PA |
|  | Linke SE, Strong DR, Myers MG, Edland SD, Hofstetter CR, Al-Delaimy WK. The relationships among physical activity, sedentary behaviour, obesity and quitting behaviours within a cohort of smokers in California. Public Health. 2016;141:232-240. doi:10.1016/j.puhe.2016.09.028 | Excluded | Non-data for outcome of interest |
|  | Loprinzi PD, Frith E. Cardiometabolic healthy obesity paradigm and all-cause mortality risk. Eur J Intern Med. 2017;43:42-45. doi:10.1016/j.ejim.2017.05.013 | Excluded | Non-data of interest according to PA |
|  | Malekshah AFT, Zaroudi M, Etemadi A, et al. The combined effects of healthy lifestyle behaviors on all-cause mortality: The Golestan cohort study. Arch Iran Med. 2016;19(11):752-761. | Excluded | Non-data of interest according to PA |
|  | Maliniak ML, Patel A V, McCullough ML, et al. Obesity, physical activity, and breast cancer survival among older breast cancer survivors in the Cancer Prevention Study-II Nutrition Cohort. Breast Cancer Res Treat. 2018;167(1):133-145. doi:10.1007/s10549-017-4470-7 | Excluded | Non-data of interest according to BMI |
|  | McTiernan A. Weight, physical activity and breast cancer survival. Proc Nutr Soc. 2018;77(4):403-411. doi:10.1017/S0029665118000010 | Excluded | Non-design |
|  | McTiernan A, Irwin M, VonGruenigen V. Weight, physical activity, diet, and prognosis in breast and gynecologic cancers. J Clin Oncol. 2010;28(26):4074-4080. doi:10.1200/JCO.2010.27.9752 | Excluded | Non-design |
|  | Messetti Christofoletti AE, Goulardins GS, Orcioli-Silva D, et al. Factors associated to mortality in adults and elderly residents in the city of Rio Claro -- SP: a cohort study. / Fatores associados à mortalidade de adultos e idosos residentes no município de Rio Claro -- SP: um estudo de coorte. Brazilian J Kineanthropometry Hum Perform. 2018;20(3):258-268. | Excluded | Non-data of interest according to PA |
|  | Meyer HE, Søgaard AJ, Tverdal A, Selmer RM. Body mass index and mortality: the influence of physical activity and smoking. Med Sci Sports Exerc. 2002;34(7):1065-1070. doi:10.1097/00005768-200207000-00002 | Excluded | Non-data of interest according to PA |
|  | Moholdt T, Lavie CJ, Nauman J. Interaction of Physical Activity and Body Mass Index on Mortality in Coronary Heart Disease: Data from the Nord-Trøndelag Health Study. Am J Med. 2017;130(8):949-957. doi:10.1016/j.amjmed.2017.01.043 | Excluded | Non-data of interest according to PA |
|  | Moore SC, Patel A V, Matthews CE, et al. Leisure time physical activity of moderate to vigorous intensity and mortality: a large pooled cohort analysis. PLoS Med. 2012;9(11):e1001335. doi:10.1371/journal.pmed.1001335 | Excluded | Non-data of interest according to PA |
|  | Navaneethan SD, Kirwan JP, Arrigain S, Schold JD. Adiposity measures, lean body mass, physical activity and mortality: NHANES 1999-2004. BMC Nephrol. 2014;15:108. doi:10.1186/1471-2369-15-108 | Excluded | Non-data of interest according to PA |
|  | Nunez C, Nair-Shalliker V, Egger S, Sitas F, Bauman A. Physical activity, obesity and sedentary behaviour and the risks of colon and rectal cancers in the 45 and up study. BMC Public Health. 2018;18(1):325. doi:10.1186/s12889-018-5225-z | Excluded | Non-data of interest according to PA |
|  | Orsini N, Bellocco R, Bottai M, Pagano M, Michaelsson K, Wolk A. Combined effects of obesity and physical activity in predicting mortality among men. J Intern Med. 2008;264(5):442-451. doi:10.1111/j.1365-2796.2008.01985.x | Excluded | Non-data of interest according to BMI |
|  | Abrahamson PE, Gammon MD, Lund MJ, et al. Recreational physical activity and survival among young women with breast cancer. Cancer. 2006;107(8):1777-1785. doi:10.1002/cncr.22201 | Excluded | Non-population |
|  | Abramowitz MK, Hall CB, Amodu A, Sharma D, Androga L, Hawkins M. Muscle mass, BMI, and mortality among adults in the United States: A population-based cohort study. PLoS One. 2018;13(4):e0194697. doi:10.1371/journal.pone.0194697 | Excluded | Non-data of interest according to PA |
|  | Abudiab M, Aijaz B, Konecny T, et al. Use of functional aerobic capacity based on stress testing to predict outcomes in normal, overweight, and obese patients. Mayo Clin Proc. 2013;88(12):1427-1434. doi:10.1016/j.mayocp.2013.10.013 | Excluded | Non-data of interest according to PA |
|  | Adams KF, Leitzmann MF, Ballard-Barbash R, et al. Body mass and weight change in adults in relation to mortality risk. Am J Epidemiol. 2014;179(2):135-144. doi:10.1093/aje/kwt254 | Excluded | Non-data of interest according to PA |
|  | Adams KF, Schatzkin A, Harris TB, et al. Overweight, obesity, and mortality in a large prospective cohort of persons 50 to 71 years old. N Engl J Med. 2006;355(8):763-778. doi:10.1056/NEJMoa055643 | Excluded | Non-data of interest according to PA |
|  | Ajani UA, Lotufo PA, Gaziano JM, et al. Body mass index and mortality among US male physicians. Ann Epidemiol. 2004;14(10):731-739. doi:10.1016/j.annepidem.2003.10.008 | Excluded | Non-data of interest according to PA |
|  | Alcazar J, Navarrete-Villanueva D, Mañas A, et al. “Fat but powerful” paradox: association of muscle power and adiposity markers with all-cause mortality in older adults from the EXERNET multicentre study. Br J Sports Med. 2021;55(21):1204-1211. doi:10.1136/bjsports-2020-103720 | Excluded | Non-data of interest according to PA |
|  | Alexander D, Allardice GM, Moug SJ, Morrison DS. A retrospective cohort study of the influence of lifestyle factors on the survival of patients undergoing surgery for colorectal cancer. Color Dis Off J Assoc Coloproctology Gt Britain Irel. 2017;19(6):544-550. doi:10.1111/codi.13594 | Excluded | Non-data of interest according to PA |
|  | Al-Shaar L, Li Y, Rimm EB, et al. Body Mass Index and Mortality Among Adults With Incident Myocardial Infarction. Am J Epidemiol. 2021;190(10):2019-2028. doi:10.1093/aje/kwab126 | Excluded | Not available data |
|  | Alvarez-Alvarez I, Zazpe I, Pérez de Rojas J, et al. Mediterranean diet, physical activity and their combined effect on all-cause mortality: The Seguimiento Universidad de Navarra (SUN) cohort. Prev Med (Baltim). 2018;106:45-52. doi:10.1016/j.ypmed.2017.09.021 | Excluded | Non-data of interest according to BMI |
|  | Amberbir A, Banda V, Singano V, et al. Effect of cardio-metabolic risk factors on all-cause mortality among HIV patients on antiretroviral therapy in Malawi: A prospective cohort study. PLoS One. 2019;14(1). doi:10.1371/journal.pone.0210629 | Excluded | Non-population |
|  | Andersen SW, Shu X-OX-O, Gao Y-T, et al. Prospective cohort study of central adiposity and risk of death in middle aged and elderly Chinese. PLoS One. 2015;10(9):e0138429. doi:10.1371/journal.pone.0138429 | Excluded | Non-data of interest according to BMI |
|  | Arem H, Pfeiffer RM, Moore SC, Brinton LA, Matthews CE. Body mass index, physical activity, and television time in relation to mortality risk among endometrial cancer survivors in the NIH-AARP Diet and Health Study cohort. Cancer Causes Control. 2016;27(11):1403-1409. doi:10.1007/s10552-016-0813-7 | Excluded | Non-data of interest according to PA |
|  | Arrieta A, Russell LB. Effects of Leisure and Non-Leisure Physical Activity on Mortality in US Adults over Two Decades. Ann Epidemiol. 2008;18(12):889-895. doi:10.1016/j.annepidem.2008.09.007 | Excluded | Non-data of interest according to BMI |
|  | Batsis JA, Mackenzie TA, Vasquez E, et al. Association of adiposity, telomere length and mortality: Data from the NHANES 1999-2002. Int J Obes. 2018;42(2):198-204. doi:10.1038/ijo.2017.202 | Excluded | Non-data of interest according to PA |
|  | Batty GD, Shipley MJ, Jarrett RJ, Breeze E, Marmot MG, Smith GD. Obesity and overweight in relation to organ-specific cancer mortality in London (UK): findings from the original Whitehall study. Int J Obes (Lond). 2005;29(10):1267-1274. doi:10.1038/sj.ijo.0803020 | Excluded | Non-data of interest according to PA |
|  | Batty GD, Shipley MJ, Kivimaki M, Marmot M, Davey Smith G. Walking pace, leisure time physical activity, and resting heart rate in relation to disease-specific mortality in London: 40 years follow-up of the original Whitehall study. An update of our work with professor Jerry N. Morris (1910-2009). Ann Epidemiol. 2010;20(9):661-669. doi:10.1016/j.annepidem.2010.03.014 | Excluded | Non-data of interest according to BMI |
|  | Bayán-Bravo A, Pérez-Tasigchana RF, López-García E, Rodríguez-Artalejo F, Guallar-Castillón P, Martínez-Gómez D. The association of major patterns of physical activity, sedentary behavior and sleeping with mortality in older adults. J Sports Sci. 2019;37(4):424-433. | Excluded | Non-data of interest according to BMI |
|  | Beleigoli AM, Boersma E, de Diniz MFH, Lima-Costa MF, Ribeiro AL. Overweight and Class I Obesity Are Associated with Lower 10-Year Risk of Mortality in Brazilian Older Adults: The Bambuí Cohort Study of Ageing. PLoS One. 2012;7(12). doi:10.1371/journal.pone.0052111 | Excluded | Non-data of interest according to PA |
|  | Beleigoli AM, Diniz MDFH, Boersma E, Silva JL, Lima-Costa MF, Ribeiro AL. The Effects of Weight and Waist Change on the Risk of Long-Term Mortality in Older Adults- The Bambuí (Brazil) Cohort Study of Aging. J Nutr Health Aging. 2017;21(8):861-866. doi:10.1007/s12603-016-0858-z | Excluded | Non-data of interest according to BMI |
|  | Beleigoli AM, Ribeiro AL, Diniz MDFH, Lima-Costa MF, Boersma E. Comparing the value of BNP in predicting mortality among community-dwelling elderly with and without overweight/obesity: The Bambuí (Brazil) Cohort Study of Aging. Int J Cardiol. 2013;168(4):4364-4366. doi:10.1016/j.ijcard.2013.05.058 | Excluded | Non-design |
|  | Bell CL, LaCroix A, Masaki K, et al. Prestroke factors associated with poststroke mortality and recovery in older women in the Women’s Health Initiative. J Am Geriatr Soc. 2013;61(8):1324-1330. doi:10.1111/jgs.12361 | Excluded | Non-data of interest according to PA |
|  | Bellocco R, Jia C, Ye W, Lagerros YT. Effects of physical activity, body mass index, waist-to-hip ratio and waist circumference on total mortality risk in the Swedish National March Cohort. Eur J Epidemiol. 2010;25(11):777-788. doi:10.1007/s10654-010-9497-6 | Excluded | Non-data of interest according to PA |
|  | Berkman LF, Syme SL. Social networks, host resistance, and mortality: a nine-year follow-up study of Alameda County residents. Am J Epidemiol. 1979;109(2):186-204. doi:10.1093/oxfordjournals.aje.a112674 | Excluded | Non-data of interest according to PA |
|  | Berrington de Gonzalez A, Hartge P, Cerhan JR, et al. Body-mass index and mortality among 1.46 million white adults. N Engl J Med. 2010;363(23):2211-2219. doi:10.1056/NEJMoa1000367 | Excluded | Non-data of interest according to PA |
|  | Bijani A, Cumming RG, Hosseini S-R, et al. Obesity paradox on the survival of elderly patients with diabetes: an AHAP-based study. J Diabetes Metab Disord. 2018;17(1):45-51. doi:10.1007/s40200-018-0337-7 | Excluded | Non-data of interest according to PA |
|  | Björck L, Novak M, Schaufelberger M, Giang KW, Rosengren A. Body weight in midlife and long-term risk of developing heart failure-a 35-year follow-up of the primary prevention study in Gothenburg, Sweden. BMC Cardiovasc Disord. 2015;15:19. doi:10.1186/s12872-015-0008-2 | Excluded | Non-data for outcome of interest |
|  | Bonaccio M, Di Castelnuovo A, Costanzo S, et al. Impact of combined healthy lifestyle factors on survival in an adult general population and in high-risk groups: prospective results from the Moli-sani Study. J Intern Med. 2019;286(2):207-220. doi:10.1111/joim.12907 | Excluded | Non-data of interest according to BMI |
|  | Boyle T, Fritschi L, Platell C, Heyworth J. Lifestyle factors associated with survival after colorectal cancer diagnosis. Br J Cancer. 2013;109(3):814-822. doi:10.1038/bjc.2013.310 | Excluded | Non-data of interest according to BMI |
|  | Boyle T, Connors JM, Gascoyne RD, et al. Physical activity, obesity and survival in diffuse large B-cell and follicular lymphoma cases. Br J Haematol. 2017;178(3):442-447. doi:10.1111/bjh.14702 | Excluded | Non-data of interest according to BMI |
|  | Buckner SL, Loenneke JP, Loprinzi PD. Lower extremity strength, systemic inflammation and all-cause mortality: Application to the “fat but fit” paradigm using cross-sectional and longitudinal designs. Physiol Behav. 2015;149:199-202. doi:10.1016/j.physbeh.2015.06.012 | Excluded | Non-design |
|  | Byberg L, Melhus H, Gedeborg R, et al. Total mortality after changes in leisure time physical activity in 50 year old men: 35 year follow-up of population based cohort. Br J Sports Med. 2009;43(7):482. | Excluded | Non-data of interest according to BMI |
|  | Cairns BJ, Balkwill A, Canoy D, Green J, Reeves GK, Beral V. Variations in vascular mortality trends, 2001-2010, among 1.3 million women with different lifestyle risk factors for the disease. Eur J Prev Cardiol. 2015;22(12):1626-1634. doi:10.1177/2047487314563710 | Excluded | Non-data of interest according to BMI |
|  | Calling S, Hedblad B, Engström G, Berglund G, Janzon L. Effects of body fatness and physical activity on cardiovascular risk: risk prediction using the bioelectrical impedance method. Scand J Public Health. 2006;34(6):568-575. doi:10.1080/14034940600595621 | Excluded | Non-population |
|  | Cesari M, Pahor M, Lauretani F, et al. Skeletal muscle and mortality results from the InCHIANTI Study. J Gerontol A Biol Sci Med Sci. 2009;64(3):377-384. doi:10.1093/gerona/gln031 | Excluded | Non-data of interest according to PA |
|  | Chaikriangkrai K, Jhun H, Graviss E, Jyothula S. Overweight-mortality paradox and impact of six-minute walk distance in lung transplantation. Ann Thorac Med. 2015;10(3):169-175. doi:10.4103/1817-1737.160835 | Excluded | Non-data of interest according to PA |
|  | Chang S-H, Pollack LM, Colditz GA. Obesity, Mortality, and Life Years Lost Associated With Breast Cancer in Nonsmoking US Women, National Health Interview Survey, 1997-2000. Prev Chronic Dis. 2013;10. doi:10.5888/pcd10.130112 | Excluded | Non-design |
|  | Chasland LC, Knuiman MW, Divitini ML, et al. Higher circulating androgens and higher physical activity levels are associated with less central adiposity and lower risk of cardiovascular death in older men. Clin Endocrinol (Oxf). 2019;90(2):375-383. doi:10.1111/cen.13905 | Excluded | Non-population |
|  | Chebet JJ, Thomson CA, Kohler LN, et al. Association of Diet Quality and Physical Activity on Obesity-Related Cancer Risk and Mortality in Black Women: Results from the Women’s Health Initiative. Cancer Epidemiol biomarkers Prev a Publ Am Assoc Cancer Res cosponsored by Am Soc Prev Oncol. 2020;29(3):591-598. doi:10.1158/1055-9965.EPI-19-1063 | Excluded | Non-data of interest according to BMI |
|  | Chen Y, Wu F, Saito E, et al. Association between type 2 diabetes and risk of cancer mortality: a pooled analysis of over 771,000 individuals in the Asia Cohort Consortium. Diabetologia. 2017;60(6):1022-1032. doi:10.1007/s00125-017-4229-z | Excluded | Non-population |
|  | Chiuve SE, Fung TT, Rexrode KM, et al. Adherence to a low-risk, healthy lifestyle and risk of sudden cardiac death among women. JAMA - J Am Med Assoc. 2011;306(1):62-69. doi:10.1001/jama.2011.907 | Excluded | Non-data of interest according to PA |
|  | Chu D-M, Wahlqvist ML, Lee M-S, Chang H-Y. Central obesity predicts non-Hodgkin’s lymphoma mortality and overall obesity predicts leukemia mortality in adult Taiwanese. J Am Coll Nutr. 2011;30(5):310-319. doi:10.1080/07315724.2011.10719974 | Excluded | Non-data of interest according to BMI |
|  | Church TS, Cheng YJ, Earnest CP, et al. Exercise capacity and body composition as predictors of mortality among men with diabetes. Diabetes Care. 2004;27(1):83-88. doi:10.2337/diacare.27.1.83 | Excluded | Non-data of interest according to PA |
|  | Church TS, LaMonte MJ, Barlow CE, Blair SN. Cardiorespiratory fitness and body mass index as predictors of cardiovascular disease mortality among men with diabetes. Arch Intern Med. 2005;165(18):2114-2120. doi:10.1001/archinte.165.18.2114 | Excluded | Non-data of interest according to PA |
|  | Clark AL, Fonarow GC, Horwich TB. Impact of cardiorespiratory fitness on the obesity paradox in patients with systolic heart failure. Am J Cardiol. 2015;115(2):209-213. doi:10.1016/j.amjcard.2014.10.023 | Excluded | Non-data of interest according to PA |
|  | Clarke AE, Carson V, Chaput J-P, et al. Meeting Canadian 24-Hour Movement Guideline recommendations and risk of all-cause mortality. Appl Physiol Nutr Metab = Physiol Appl Nutr Metab. 2021;46(12):1487-1494. doi:10.1139/apnm-2021-0010 | Excluded | Non-population |
|  | Courneya KS, Segal RJ, McKenzie DC, et al. Effects of exercise during adjuvant chemotherapy on breast cancer outcomes. Med Sci Sports Exerc. 2014;46(9):1744-1751. doi:10.1249/MSS.0000000000000297 | Excluded | Non-design |
|  | Crespo CJ, Garcia-Palmieri MR, Smit E, et al. Physical activity and prostate cancer mortality in Puerto Rican men. J Phys Act Health. 2008;5(6):918-929. doi:10.1123/jpah.5.6.918 | Excluded | Data in previous included |
|  | Croci I, Coombes JS, Sandbakk SB, et al. Non-alcoholic fatty liver disease: prevalence and all-cause mortality according to sedentary behaviour and cardiorespiratory fitness. The HUNT Study. Prog Cardiovasc Dis. Published online 2019:#pagerange#. doi:10.1016/J.PCAD.2019.01.005 | Excluded | Non-data of interest according to BMI |
|  | Crump C, Sundquist J, Winkleby MA, Sundquist K. Interactive Effects of Aerobic Fitness, Strength, and Obesity on Mortality in Men. Am J Prev Med. 2017;52(3):353-361. doi:10.1016/j.amepre.2016.10.002 | Excluded | Non-data of interest according to PA |
|  | Danon-Hersch N, Fustinoni S, Bovet P, Spagnoli J, Santos-Eggimann B. Association between Adiposity and disability in the Lc65+ Cohort. J Nutr Health Aging. 2017;21(7):799-810. doi:10.1007/s12603-016-0813-z | Excluded | Non-data of interest according to PA |
|  | Davidson LE, Hunt SC, Adams TD. Fitness versus adiposity in cardiovascular disease risk. Eur J Clin Nutr. 2019;73(2):225-230. doi:10.1038/s41430-018-0333-5 | Excluded | Non-design |
|  | Davos CH, Doehner W, Rauchhaus M, et al. Body mass and survival in patients with chronic heart failure without cachexia: the importance of obesity. J Card Fail. 2003;9(1):29-35. doi:10.1054/jcaf.2003.4 | Excluded | Non-data of interest according to PA |
|  | De Schutter A, Lavie CJ, Patel DA, Artham SM, Milani R V. Relation of body fat categories by Gallagher classification and by continuous variables to mortality in patients with coronary heart disease. Am J Cardiol. 2013;111(5):657-660. doi:10.1016/j.amjcard.2012.11.013 | Excluded | Non-data of interest according to PA |
|  | Dehal A, Garrett T, Tedders SH, Arroyo C, Afriyie-Gyawu E, Zhang J. Body Mass Index and Death Rate of Colorectal Cancer Among a National Cohort of U.S. Adults. Nutr CANCER-AN Int J. 2011;63(8):1218-1225. doi:10.1080/01635581.2011.607539 | Excluded | Non-data of interest according to PA |
|  | Del Brutto OH, Mera RM, Del Brutto VJ. Nonfatal Stroke and All-Cause Mortality among Community-Dwelling Older Adults Living in Rural Ecuador: A Population-Based, Prospective Study. J Neurosci Rural Pract. 2018;9(4):551-555. doi:10.4103/jnrp.jnrp_79_18 | Excluded | Non-data of interest according to PA |
|  | Dhalwani NN, Zaccardi F, Davies MJ, Khunti K. Body mass index and mortality in people with and without diabetes: A UK Biobank study. Nutr Metab Cardiovasc Dis. 2018;28(12):1208-1216. doi:10.1016/j.numecd.2018.07.007 | Excluded | Non-data of interest according to PA |
|  | Dhana K, Berghout MA, Peeters A, et al. Obesity in older adults and life expectancy with and without cardiovascular disease. Int J Obes (Lond). 2016;40(10):1535-1540. doi:10.1038/ijo.2016.94 | Excluded | Non-data of interest according to PA |
|  | Diehr P, Bild DE, Harris TB, Duxbury A, Siscovick D, Rossi M. Body Mass Index and Mortality in Nonsmoking Older Adults: The Cardiovascular Health Study. Am J Public Health. 1998;88(4):623-629. | Excluded | Non-data of interest according to PA |
|  | Divo MJ, Cabrera C, Casanova C, et al. Comorbidity Distribution, Clinical Expression and Survival in COPD Patients with Different Body Mass Index. Chronic Obstr Pulm Dis (Miami, Fla). 2014;1(2):229-238. doi:10.15326/jcopdf.1.2.2014.0117 | Excluded | Non-data of interest according to PA |
|  | Dobson A, McLaughlin D, Almeida O, et al. Impact of behavioural risk factors on death within 10 years for women and men in their 70s: absolute risk charts. BMC Public Health. 2012;12:669. doi:10.1186/1471-2458-12-669 | Excluded | Non-data of interest according to BMI |
|  | Dolan CM, L. Kelsey J, Kraemer H, Browner W, Ensrud K. Associations Between Body Composition, Anthropometry, and Mortality in Women Aged 65 Years and Older. Am J Public Health. 2007;97(5):913-918. | Excluded | Non-data of interest according to PA |
|  | Dorn JP, Cerny FJ, Epstein LH, et al. Work and leisure time physical activity and mortality in men and women from a general population sample. Ann Epidemiol. 1999;9(6):366-373. doi:10.1016/s1047-2797(99)00025-3 | Excluded | Non-population |
|  | Duong T V, Wong T-C, Chen H-H, et al. Impact of percent body fat on all-cause mortality among adequate dialysis patients with and without insulin resistance: A multi-center prospective cohort study. Nutrients. 2019;11(6). doi:10.3390/nu11061304 | Excluded | Non-population |
|  | Eguchi E, Iso H, Tanabe N, Yatsuya H, Tamakoshi A. Is the association between healthy lifestyle behaviors and cardiovascular mortality modified by overweight status? The Japan Collaborative Cohort Study. Prev Med (Baltim). 2014;62:142-147. doi:10.1016/j.ypmed.2013.12.004 | Excluded | Non-data of interest according to PA |
|  | Eilat-Adar S, Goldbourt U, Resnick HE, Howard B V. Intentional weight loss, blood lipids and coronary morbidity and mortality. Curr Opin Lipidol. 2005;16(1):5-9. doi:10.1097/00041433-200502000-00003 | Excluded | Non-design |
|  | Elme A, Utriainen M, Kellokumpu-Lehtinen P, et al. Obesity and physical inactivity are related to impaired physical health of breast cancer survivors. Anticancer Res. 2013;33(4):1595-1602. | Excluded | Non-data for outcome of interest |
|  | Etemadi A, Abnet CC, Kamangar F, et al. Impact of body size and physical activity during adolescence and adult life on overall and cause-specific mortality in a large cohort study from Iran. Eur J Epidemiol. 2014;29(2):95-109. doi:10.1007/s10654-014-9883-6 | Excluded | Non-data of interest according to BMI |
|  | Fardman A, Banschick GD, Rabia R, et al. Cardiorespiratory fitness and survival following cancer diagnosis. Eur J Prev Cardiol. 2021;28(11):1242-1249. doi:10.1177/2047487320930873 | Excluded | Non-data of interest according to BMI |
|  | Farrell SW, Braun L, Barlow CE, Cheng YJ, Blair SN. The relation of body mass index, cardiorespiratory fitness, and all-cause mortality in women. Obes Res. 2002;10(6):417-423. doi:10.1038/oby.2002.58 | Excluded | Non-data of interest according to PA |
|  | Farrell SW, Barlow CE, Willis BL, et al. Cardiorespiratory Fitness, Different Measures of Adiposity, and Cardiovascular Disease Mortality Risk in Women. J Womens Health (Larchmt). 2020;29(3):319-326. doi:10.1089/jwh.2019.7793 | Excluded | Non-data of interest according to PA |
|  | Farrell SW, Cortese GM, LaMonte MJ, Blair SN. Cardiorespiratory fitness, different measures of adiposity, and cancer mortality in men. Obesity (Silver Spring). 2007;15(12):3140-3149. doi:10.1038/oby.2007.374 | Excluded | Non-data of interest according to PA |
|  | Farrell SW, Finley CE, Jackson AW, Vega GL, Morrow JRJ. Association of multiple adiposity exposures and cardiorespiratory fitness with all-cause mortality in men: the Cooper Center Longitudinal Study. Mayo Clin Proc. 2014;89(6):772-780. doi:10.1016/j.mayocp.2014.03.012 | Excluded | Non-data of interest according to PA |
|  | Farrell SW, Finley CE, McAuley PA, Frierson GM. Cardiorespiratory fitness, different measures of adiposity, and total cancer mortality in women. Obesity (Silver Spring). 2011;19(11):2261-2267. doi:10.1038/oby.2010.345 | Excluded | Non-data of interest according to PA |
|  | Farrell SW, Finley CE, Radford NB, Haskell WL. Cardiorespiratory fitness, body mass index, and heart failure mortality in men: Cooper Center Longitudinal Study. Circ Heart Fail. 2013;6(5):898-905. doi:10.1161/CIRCHEARTFAILURE.112.000088 | Excluded | Non-data of interest according to PA |
|  | Farrell SW, Fitzgerald SJ, McAuley PA, Barlow CE. Cardiorespiratory fitness, adiposity, and all-cause mortality in women. Med Sci Sports Exerc. 2010;42(11):2006-2012. doi:10.1249/MSS.0b013e3181df12bf | Excluded | Non-data of interest according to PA |
|  | Faselis C, Doumas M, Panagiotakos D, et al. Body mass index, exercise capacity, and mortality risk in male veterans with hypertension. Am J Hypertens. 2012;25(4):444-450. doi:10.1038/ajh.2011.242 | Excluded | Non-data of interest according to PA |
|  | Fleary SA, Mehl R, Nigg C. Predicting Hawaiian Youth’s Physical Activity and Fruit and Vegetable Consumption Behaviors: A 10-Year Cohort Study. J Phys Act Health. 2019;16(8):644-646. | Excluded | Non-data of interest according to BMI |
|  | Fleenor BS, Peterman JE, Kaminsky LA, Whaley MH, Harber MP. The Association Between Pulse Pressure And All-cause Mortality Is Dependent On Cardiorespiratory Fitness. Med Sci Sport Exerc. 2021;53:444. | Excluded | Non-data for outcome of interest |
|  | Fraser GE, Sumbureru D, Pribis P, Neil RL, Frankson MA. Association among health habits, risk factors, and all-cause mortality in a black California population. Epidemiology. 1997;8(2):168-174. doi:10.1097/00001648-199703000-00008 | Excluded | Non-data of interest according to PA |
|  | Freedman DS. Clustering of coronary heart disease risk factors among obese children. J Pediatr Endocrinol Metab. 2002;15(8):1099-1108. doi:10.1515/JPEM.2002.15.8.1099 | Excluded | Non-population |
|  | Garnvik LE, Malmo V, Janszky I, Wisløff U, Loennechen JP, Nes BM. Physical activity modifies the risk of atrial fibrillation in obese individuals: The HUNT3 study. Eur J Prev Cardiol. 2018;25(15):1646-1652. doi:10.1177/2047487318784365 | Excluded | Non-data of interest according to PA |
|  | Genkinger JM, Kitahara CM, Bernstein L, et al. Central adiposity, obesity during early adulthood, and pancreatic cancer mortality in a pooled analysis of cohort studies. Ann Oncol Off J Eur Soc Med Oncol. 2015;26(11):2257-2266. doi:10.1093/annonc/mdv355 | Excluded | Non-design |
|  | Ghazizadeh H, Mirinezhad SMR, Asadi Z, et al. Association between obesity categories with cardiovascular disease and its related risk factors in the MASHAD cohort study population. J Clin Lab Anal. 2020;34(5). doi:10.1002/jcla.23160 | Excluded | Non-data of interest according to PA |
|  | Gislefoss RE, Stenehjem JS, Hektoen HH, et al. Vitamin D, obesity and leptin in relation to bladder cancer incidence and survival: prospective protocol study. BMJ Open. 2018;8(3):e019309. doi:10.1136/bmjopen-2017-019309 | Excluded | Non-design |
|  | Golabi P, Paik JM, Arshad T, Younossi Y, Mishra A, Younossi ZM. Mortality of NAFLD According to the Body Composition and Presence of Metabolic Abnormalities. Hepatol Commun. 2020;4(8):1136-1148. doi:10.1002/hep4.1534 | Excluded | Non-data of interest according to PA |
|  | Grace MS, Lynch BM, Dillon F, Barr ELM, Owen N, Dunstan DW. Joint associations of smoking and television viewing time on cancer and cardiovascular disease mortality. Int J cancer. 2017;140(7):1538-1544. doi:10.1002/ijc.30580 | Excluded | Non-data of interest according to BMI |
|  | Graff-Iversen S, Selmer R, Skurtveit S, Sørensen M. Occupational physical activity, overweight, and mortality: A follow-up study of 47,405 norwegian women and men. Res Q Exerc Sport. 2007;78(3):151-161. doi:10.1080/02701367.2007.10599412 | Excluded | Non-data of interest according to BMI |
|  | Grundy SM, Blackburn G, Higgins M, Lauer R, Perri MG, Ryan D. Physical activity in the prevention and treatment of obesity and its comorbidities. Med Sci Sports Exerc. 1999;31(11):S502-S508. doi:10.1097/00005768-199911001-00003 | Excluded | Non-design |
|  | Guercio BJ, Zhang S, Ou F-S, et al. Associations of Physical Activity With Survival and Progression in Metastatic Colorectal Cancer: Results From Cancer and Leukemia Group B (Alliance)/SWOG 80405. J Clin Oncol. 2019;37(29):2620-+. doi:10.1200/JCO.19.01019 | Excluded | Non-data of interest according to BMI |
|  | Håheim LL, Tonstad S, Hjermann I, Leren P, Holme I. Predictiveness of body mass index for fatal coronary heart disease in men according to length of follow-up: a 21-year prospective cohort study. Scand J Public Health. 2007;35(1):4-10. doi:10.1080/14034940510032293 | Excluded | Non-data of interest according to BMI |
|  | Halldin A-K, Lissner L, Lernfelt B, Björkelund C. Impact of changes in physical activity or BMI on risk of heart failure in women - the prospective population study of women in Gothenburg. Scand J Prim Health Care. 2020;38(1):56-65. doi:10.1080/02813432.2020.1717083 | Excluded | Non-data of interest according to PA |
|  | Hamer M, Ding D, Chau J, Duncan MJ, Stamatakis E. Association between TV viewing and heart disease mortality: observational study using negative control outcome. J Epidemiol Community Health. 2020;74(4):391-394. doi:10.1136/jech-2019-212739 | Excluded | Non-data of interest according to BMI |
|  | 1081. Hamer M, O’Donovan G, Stamatakis E. Lifestyle risk factors, obesity and infectious disease mortality in the general population: Linkage study of 97,844 adults from England and Scotland. Prev Med (Baltim). 2019;123:65-70. doi:10.1016/j.ypmed.2019.03.002 | Excluded | Non-data of interest according to BMI |
|  | 1082. Hamilton MT, Hamilton DG, Zderic TW. Role of low energy expenditure and sitting in obesity, metabolic syndrome, type 2 diabetes, and cardiovascular disease. Diabetes. 2007;56(11):2655-2667. doi:10.2337/db07-0882 | Excluded | Non-data for outcome of interest |
|  | 1083. Hammer MD, Andersen AJ, Larsen SC, Simonsen MK, Heitmann BL. The association between general and central obesity and the risks of coronary heart disease in women with and without a familial predisposition to obesity: findings from the Danish Nurse Cohort. Int J Obes (Lond). 2022;46(2):433-436. doi:10.1038/s41366-021-00990-4 | Excluded | Non-data of interest according to PA |
|  | 1095. Hardee JP, Porter RR, Sui X, et al. The effect of resistance exercise on all-cause mortality in cancer survivors. Mayo Clin Proc. 2014;89(8):1108-1115. doi:10.1016/j.mayocp.2014.03.018 | Excluded | Non-data of interest according to BMI |
|  | 1127. Heir T, Erikssen J, Sandvik L. Overweight as predictor of long-term mortality among healthy, middle-aged men: A prospective cohort study. Prev Med (Baltim). 2011;52(3-4):223-226. doi:10.1016/j.ypmed.2011.01.010 | Excluded | Non-population |
|  | 1132. Hemmingsson E, Väisänen D, Andersson G, Wallin P, Ekblom-Bak E. Combinations of BMI and cardiorespiratory fitness categories: trends between 1995 and 2020 and associations with CVD incidence and mortality and all-cause mortality in 471 216 adults. Eur J Prev Cardiol. Published online October 2021. doi:10.1093/eurjpc/zwab169 | Excluded | Non-data of interest according to PA |
|  | 1156. Hershey MS, Martínez-González MÁ, Álvarez-Álvarez I, Martínez Hernández JA, Ruiz-Canela M. The Mediterranean diet and physical activity: better together than apart for the prevention of premature mortality. Br J Nutr. Published online August 2021:1-12. doi:10.1017/S0007114521002877 | Excluded | Non-data of interest according to BMI |
|  | 1190. Horwich TB, Broderick S, Chen L, et al. Relation among body mass index, exercise training, and outcomes in chronic systolic heart failure. Am J Cardiol. 2011;108(12):1754-1759. doi:10.1016/j.amjcard.2011.07.051 | Excluded | Non-design |
|  | 1198. Hu FB, Willett WC, Li T, Stampfer MJ, Colditz GA, Manson JE. Adiposity as compared with physical activity in predicting mortality among women. N Engl J Med. 2004;351(26):2694-2703. doi:10.1056/NEJMoa042135 | Excluded | Non-data of interest according to PA |
|  | 1200. Hu G, Tuomilehto J, Silventoinen K, Barengo N, Jousilahti P. Joint effects of physical activity, body mass index, waist circumference and waist-to-hip ratio with the risk of cardiovascular disease among middle-aged Finnish men and women. Eur Heart J. 2004;25(24):2212-2219. doi:10.1016/j.ehj.2004.10.020 | Excluded | Non-data of interest according to PA |
|  | 1203. Huang T, Eliassen AH, Hankinson SE, et al. A prospective study of leisure-time physical activity and risk of incident epithelial ovarian cancer: Impact by menopausal status. Int J Cancer. 2016;138(4):843-852. doi:10.1002/ijc.29834 | Excluded | Non-data of interest according to BMI |
|  | 1204. Huang YY, Jiang CQ, Xu L, et al. Adiposity change and mortality in middle-aged to older Chinese: An 8-year follow-up of the Guangzhou Biobank Cohort Study. BMJ Open. 2020;10(12). doi:10.1136/bmjopen-2020-039239 | Excluded | Non-data of interest according to PA |
|  | 1205. Huang Y, Jiang C, Xu L, et al. Mortality in relation to changes in physical activity in middle-aged to older Chinese: An 8-year follow-up of the Guangzhou Biobank Cohort Study. J Sport Heal Sci. 2021;10(4):430-438. doi:10.1016/j.jshs.2020.08.007 | Excluded | Non-data of interest according to BMI |
|  | 1231. Inoue M, Iso H, Yamamoto S, et al. Daily total physical activity level and premature death in men and women: results from a large-scale population-based cohort study in Japan (JPHC study). Ann Epidemiol. 2008;18(7):522-530. doi:10.1016/j.annepidem.2008.03.008 | Excluded | Non-data of interest according to BMI |
|  | 1247. Jae SY, Franklin BA, Kurl S, et al. Effect of Cardiorespiratory Fitness on Risk of Sudden Cardiac Death in Overweight/Obese Men Aged 42 to 60 Years. Am J Cardiol. 2018;122(5):775-779. doi:10.1016/j.amjcard.2018.05.017 | Excluded | Non-data of interest according to BMI |
|  | 1259. Janssen I. Morbidity and mortality risk associated with an overweight BMI in older men and women. Obesity (Silver Spring). 2007;15(7):1827-1840. doi:10.1038/oby.2007.217 | Excluded | Non-data of interest according to PA |
|  | 1324. Kamil-Rosenberg S, Kokkinos P, Grune de Souza e Silva C, et al. Association between cardiorespiratory fitness, obesity, and incidence of atrial fibrillation. IJC Hear Vasc. 2020;31. doi:10.1016/j.ijcha.2020.100663 | Excluded | Non-data for outcome of interest |
|  | 1355. Katzmarzyk PT, Craig CL. Independent effects of waist circumference and physical activity on all-cause mortality in Canadian women. Appl Physiol Nutr Metab. 2006;31(3):271-276. doi:10.1139/H05-038 | Excluded | Non-data of interest according to BMI |
|  | 1356. Katzmarzyk PT, Janssen I, Ardern CI. Physical inactivity, excess adiposity and premature mortality. Obes Rev an Off J Int Assoc Study Obes. 2003;4(4):257-290. doi:10.1046/j.1467-789x.2003.00120.x | Excluded | Non-design |
|  | 1359. Katzmarzyk PT, Church TS, Janssen I, Ross R, Blair SN. Metabolic syndrome, obesity, and mortality: impact of cardiorespiratory fitness. Diabetes Care. 2005;28(2):391-397. doi:10.2337/diacare.28.2.391 | Excluded | Non-data of interest according to PA |
|  | 1414. Kim NH, Seo JHA, Cho H, et al. Risk of the Development of Diabetes and Cardiovascular Disease in Metabolically Healthy Obese People. Med (United States). 2016;95(15):e3384. doi:10.1097/MD.0000000000003384 | Excluded | Non-data of interest according to PA |
|  | 1416. Kim Y-H, Kim SM, Han K, et al. Change in Weight and Body Mass Index Associated With All-Cause Mortality in Korea: A Nationwide Longitudinal Study. J Clin Endocrinol Metab. 2017;102(11):4041-4050. doi:10.1210/jc.2017-00787 | Excluded | Non-data of interest according to PA |
|  | 1478. Køster-Rasmussen R, Simonsen MK, Siersma V, Henriksen JE, Heitmann BL, de Fine Olivarius N. Intentional Weight Loss and Longevity in Overweight Patients with Type 2 Diabetes: A Population-Based Cohort Study. PLoS One. 2016;11(1):e0146889. doi:10.1371/journal.pone.0146889 | Excluded | Non-data of interest according to PA |
|  | 1495. Kuiper JG, Phipps AI, Neuhouser ML, et al. Recreational physical activity, body mass index, and survival in women with colorectal cancer. Cancer Causes Control. 2012;23(12):1939-1948. doi:10.1007/s10552-012-0071-2 | Excluded | Non-data for outcome of interest |
|  | 1528. LaMonte MJ, Blair SN. Physical activity, cardiorespiratory fitness, and adiposity: contributions to disease risk. Curr Opin Clin Nutr Metab Care. 2006;9(5):540-546. doi:10.1097/01.mco.0000241662.92642.08 | Excluded | Non-design |
|  | 1536. Lapidus L, Bengtsson C. Socioeconomic factors and physical activity in relation to cardiovascular disease and death. A 12 year follow up of participants in a population study of women in Gothenburg, Sweden. Br Heart J. 1986;55(3):295-301. doi:10.1136/hrt.55.3.295 | Excluded | Non-data of interest according to BMI |
|  | 1537. Lapidus L, Andersson SW, Bengtsson C, Björkelund C, Rossander-Hulthén L, Lissner L. Weight and length at birth and their relationship to diabetes incidence and all-cause mortality--a 32-year follow-up of the population study of women in Gothenburg, Sweden. Prim Care Diabetes. 2008;2(3):127-133. doi:10.1016/j.pcd.2008.05.002 | Excluded | Non-data of interest according to PA |
|  | 1547. Laukkanen JA, Lakka TA, Rauramaa R, et al. Cardiovascular fitness as a predictor of mortality in men. Arch Intern Med. 2001;161(6):825-831. doi:10.1001/archinte.161.6.825 | Excluded | Non-data for outcome of interest |
|  | 2351. Rosella LC, Kornas K, Huang A, Grant L, Bornbaum C, Henry D. Population risk and burden of health behavioral-related all-cause, premature, and amenable deaths in Ontario, Canada: Canadian Community Health Survey-linked mortality files. Ann Epidemiol. 2019;32:49-57.e3. doi:10.1016/j.annepidem.2019.01.009 | Excluded | Non-data for outcome of interest |
|  | 2352. Rosen P, Dohrn I, Hagströmer M. Association between physical activity and all‐cause mortality: A 15‐year follow‐up using a compositional data analysis. Scand J Med Sci Sports. 2020;30(1):100-107. | Excluded | Non-data of interest according to BMI |
|  | 2355. Rosengren A, Wedel H, Wilhelmsen L. Body weight and weight gain during adult life in men in relation to coronary heart disease and mortality. A prospective population study. Eur Heart J. 1999;20(4):269-277. | Excluded | Non-data for outcome of interest |
|  | 2359. Rossi AP, Fantin F, Caliari C, et al. Dynapenic abdominal obesity as predictor of mortality and disability worsening in older adults: A 10-year prospective study. Clin Nutr. 2016;35(1):199-204. doi:10.1016/j.clnu.2015.02.005 | Excluded | Non-data of interest according to PA |
|  | 2373. Rozanski A, Gransar H, Hayes SW, et al. Synergistic Assessment of Mortality Risk According to Body Mass Index and Exercise Ability and Capacity in Patients Referred for Radionuclide Stress Testing. Mayo Clin Proc. 2021;96(12):3001-3011. doi:10.1016/j.mayocp.2021.05.021 | Excluded | Non-data of interest according to PA |
|  | 2378. Ruiz-Casado A, Verdugo AS, Solano MJO, et al. Objectively Assessed Physical Activity Levels in Spanish Cancer Survivors. Oncol Nurs Forum. 2014;41(1):E12-E20. doi:10.1188/14.ONF.E12-E20 | Excluded | Non-data for outcome of interest |
|  | 2382. Russ TC, Lee I-M, Sesso HD, Muniz-Terrera G, Batty GD. Five-decade trajectories in body mass index in relation to dementia death: follow-up of 33,083 male Harvard University alumni. Int J Obes (Lond). 2019;43(9):1822-1829. doi:10.1038/s41366-018-0274-z | Excluded | Non-data of interest according to PA |
|  | 2393. Sabbag A, Mazin I, Rott D, et al. The prognostic significance of improvement in exercise capacity in heart failure patients who participate in cardiac rehabilitation programme. Eur J Prev Cardiol. 2018;25(4):354-361. doi:10.1177/2047487317750427 | Excluded | Non-data of interest according to BMI |
|  | 2469. Schnohr C, Højbjerre L, Riegels M, et al. Does educational level influence the effects of smoking, alcohol, physical activity, and obesity on mortality? A prospective population study. Scand J Public Health. 2004;32(4):250-256. doi:10.1080/14034940310019489 | Excluded | Non-data for outcome of interest |
|  | 2474. Schooling CM, Lam TH, Li Z Bin, et al. Obesity, physical activity, and mortality in a prospective chinese elderly cohort. Arch Intern Med. 2006;166(14):1498-1504. doi:10.1001/archinte.166.14.1498 | Excluded | Non-data for outcome of interest |
|  | 2553. Sierra-Johnson J, Wright SR, Lopez-Jimenez F, Allison TG. Relation of body mass index to fatal and nonfatal cardiovascular events after cardiac rehabilitation. Am J Cardiol. 2005;96(2):211-214. doi:10.1016/j.amjcard.2005.03.046 | Excluded | Non-data of interest according to PA |
|  | 2565. Simon TG, Kim MN, Luo X, et al. Physical activity compared to adiposity and risk of liver-related mortality: Results from two prospective, nationwide cohorts. J Hepatol. 2020;72(6):1062-1069. doi:10.1016/j.jhep.2019.12.022 | Excluded | Non-data of interest according to PA |
|  | 2571. Singh PN, Clark RW, Herring P, Sabaté J, Shavlik D, Fraser GE. Obesity and life expectancy among long-lived Black adults. J Gerontol A Biol Sci Med Sci. 2014;69(1):63-72. doi:10.1093/gerona/glt049 | Excluded | Non-data of interest according to PA |
|  | 2608. Song M, Giovannucci E. Preventable Incidence and Mortality of Carcinoma Associated With Lifestyle Factors Among White Adults in the United States. JAMA Oncol. 2016;2(9):1154-1161. doi:10.1001/jamaoncol.2016.0843 | Excluded | Non-data for outcome of interest |
|  | 2635. Staiano AE, Reeder BA, Elliott S, et al. Physical activity level, waist circumference, and mortality. Appl Physiol Nutr Metab = Physiol Appl Nutr Metab. 2012;37(5):1008-1013. doi:10.1139/h2012-058 | Excluded | Non-data of interest according to BMI |
|  | 2636. Stamatakis E, Hamer M, Dunstan DW. Screen-based entertainment time, all-cause mortality, and cardiovascular events: population-based study with ongoing mortality and hospital events follow-up. J Am Coll Cardiol. 2011;57(3):292-299. doi:10.1016/j.jacc.2010.05.065 | Excluded | Non-data of interest according to BMI |
|  | 2647. Stenholm S, Mehta NK, Elo IT, Heliövaara M, Koskinen S, Aromaa A. Obesity and muscle strength as long-term determinants of all-cause mortality--a 33-year follow-up of the Mini-Finland Health Examination Survey. Int J Obes (Lond). 2014;38(8):1126-1132. doi:10.1038/ijo.2013.214 | Excluded | Non-data of interest according to PA |
|  | 2656. Stevens J, Evenson KR, Thomas O, Cai J, Thomas R. Associations of fitness and fatness with mortality in Russian and American men in the lipids research clinics study. Int J Obes. 2004;28(11):1463-1470. doi:10.1038/sj.ijo.0802770 | Excluded | Non-data of interest according to PA |
|  | 2658. Stevens J, Cai J, Evenson KR, Thomas R. Fitness and fatness as predictors of mortality from all causes and from cardiovascular disease in men and women in the lipid research clinics study. Am J Epidemiol. 2002;156(9):832-841. doi:10.1093/aje/kwf114 | Excluded | Non-data of interest according to BMI |
|  | 2679. Stringhini S, Carmeli C, Jokela M, et al. Socioeconomic status and the 25 × 25 risk factors as determinants of premature mortality: a multicohort study and meta-analysis of 1·7 million men and women. Lancet (London, England). 2017;389(10075):1229-1237. doi:10.1016/S0140-6736(16)32380-7 | Excluded | Non-data for outcome of interest |
|  | 2690. Suadicani P, Hein HO, von Eyben FE, Gyntelberg F. Metabolic and lifestyle predictors of ischemic heart disease and all-cause mortality among normal weight, overweight, and obese men: a 16-year follow-up in the Copenhagen Male Study. Metab Syndr Relat Disord. 2009;7(2):97-104. doi:10.1089/met.2008.0041 | Excluded | Non-data of interest according to PA |
|  | 2695. Sui X, LaMonte MJ, Laditka JN, et al. Cardiorespiratory fitness and adiposity as mortality predictors in older adults. JAMA. 2007;298(21):2507-2516. doi:10.1001/jama.298.21.2507 | Excluded | Non-data of interest according to PA |
|  | 2696. Sui X, Li H, Zhang J, Chen L, Zhu L, Blair SN. Percentage of deaths attributable to poor cardiovascular health lifestyle factors: Findings from the Aerobics Center Longitudinal Study. Epidemiol Res Int. 2013;2013. doi:10.1155/2013/437465 | Excluded | Non-data for outcome of interest |
|  | 2701. Sun H, Ren X, Chen Z, et al. Association between body mass index and mortality in a prospective cohort of Chinese adults. Medicine (Baltimore). 2016;95(32):e4327. doi:10.1097/MD.0000000000004327 | Excluded | Non-data of interest according to PA |
|  | 2702. Sun L, Zhuang L-P, Li X-Z, Zheng J, Wu W-F. Tai Chi can prevent cardiovascular disease and improve cardiopulmonary function of adults with obesity aged 50 years and older: A long-term follow-up study. Medicine (Baltimore). 2019;98(42):e17509. doi:10.1097/MD.0000000000017509 | Excluded | Non-design |
|  | 2703. Sun Q, Townsend MK, Okereke OI, Franco OH, Hu FB, Grodstein F. Adiposity and weight change in mid-life in relation to healthy survival after age 70 in women: prospective cohort study. BMJ. 2009;339:b3796. doi:10.1136/bmj.b3796 | Excluded | Non-data of interest according to PA |
|  | 2705. Sung K-C, Ryu S, Cheong ES, et al. All-Cause and Cardiovascular Mortality Among Koreans: Effects of Obesity and Metabolic Health. Am J Prev Med. 2015;49(1):62-71. doi:10.1016/j.amepre.2015.02.010 | Excluded | Non-data of interest according to PA |
|  | 2711. Suzuki T, Kohro T, Hayashi D, Yamazaki T, Nagai R. Frequency and impact of lifestyle modification in patients with coronary artery disease: the Japanese Coronary Artery Disease (JCAD) study. Am Heart J. 2012;163(2):268-273. doi:10.1016/j.ahj.2011.10.014 | Excluded | Non-data of interest according to BMI |
|  | 2719. Sydó N, Merkely B, Carta KAG, et al. Effect of Cardiorespiratory Fitness on Co-Morbidities and Mortality in Never, Past, and Current Smokers. Am J Cardiol. 2018;122(10):1765-1772. doi:10.1016/j.amjcard.2018.08.012 | Excluded | Non-data of interest according to BMI |
|  | 2720. Sydó N, Sydó T, Gonzalez Carta KA, et al. Prognostic Performance of Heart Rate Recovery on an Exercise Test in a Primary Prevention Population. J Am Heart Assoc. 2018;7(7). doi:10.1161/JAHA.117.008143 | Excluded | Non-data for outcome of interest |
|  | 2721. Sydó N, Sydó T, Gonzalez Carta KA, et al. Significance of an Increase in Diastolic Blood Pressure During a Stress Test in Terms of Comorbidities and Long-Term Total and CV Mortality. Am J Hypertens. 2018;31(9):976-980. doi:10.1093/ajh/hpy080 | Excluded | Non-data for outcome of interest |
|  | 2737. Tanaka S, Tanaka S, Iimuro S, et al. Body mass index and mortality among Japanese patients with type 2 diabetes: pooled analysis of the Japan diabetes complications study and the Japanese elderly diabetes intervention trial. J Clin Endocrinol Metab. 2014;99(12):E2692-6. doi:10.1210/jc.2014-1855 | Excluded | Non-data of interest according to PA |
|  | 2739. Tarp J, Grøntved A, Sanchez-Lastra MA, Dalene KE, Ding D, Ekelund U. Fitness, Fatness, and Mortality in Men and Women From the UK Biobank: Prospective Cohort Study. J Am Heart Assoc. 2021;10(6):e019605. doi:10.1161/JAHA.120.019605 | Excluded | Non-data of interest according to PA |
|  | 2759. Thomson CA, Crane TE, Wertheim BC, et al. Diet quality and survival after ovarian cancer: Results from the Women’s Health Initiative. J Natl Cancer Inst. 2014;106(11). doi:10.1093/jnci/dju314 | Excluded | Non-data for outcome of interest |
|  | 2761. Thornqvist C, Gislason GH, Køber L, Jensen PF, Torp-Pedersen C, Andersson C. Body mass index and risk of perioperative cardiovascular adverse events and mortality in 34,744 Danish patients undergoing hip or knee replacement. Acta Orthop. 2014;85(5):456-462. | Excluded | Non-data for outcome of interest |
|  | 2764. Thorpe RJJ, Wilson-Frederick SM, Bowie J V, et al. Health behaviors and all-cause mortality in African American men. Am J Mens Health. 2013;7(4 Suppl):8S-18S. doi:10.1177/1557988313487552 | Excluded | Non-data for outcome of interest |
|  | 2774. Tikkanen-Dolenc H, Wadén J, Forsblom C, et al. Physical Activity Reduces Risk of Premature Mortality in Patients With Type 1 Diabetes With and Without Kidney Disease. Diabetes Care. 2017;40(12):1727-1732. doi:10.2337/dc17-0615 | Excluded | Non-data of interest according to BMI |
|  | 2803. Tsugane S, Sasaki S, Tsubono Y. Under- and overweight impact on mortality among middle-aged Japanese men and women: a 10-y follow-up of JPHC study cohort I. Int J Obes Relat Metab Disord J Int Assoc Study Obes. 2002;26(4):529-537. doi:10.1038/sj.ijo.0801961 | Excluded | Non-data of interest according to BMI |
|  | 2813. Tupper OD, Andersen ZJ, Ulrik CS. Demographic, lifestyle and comorbid risk factors for all-cause mortality in a Danish cohort of middle-aged adults with incident asthma. BMJ Open. 2021;11(10). doi:10.1136/bmjopen-2021-049243 | Excluded | Non-data for outcome of interest |
|  | 2822. Uretsky S, Supariwala A, Gurram S, et al. The interaction of exercise ability and body mass index upon long-term outcomes among patients undergoing stress-rest perfusion single-photon emission computed tomography imaging. Am Heart J. 2013;166(1):127-133. doi:10.1016/j.ahj.2013.03.027 | Excluded | Non-data of interest according to PA |
|  | 2831. Vainshelboim B, Chan K, Chen Z, Myers J. Cardiorespiratory fitness and cancer in men with cardiovascular disease: Analysis from the Veterans Exercise Testing Study. Eur J Prev Cardiol. 2021;28(7):715-721. doi:10.1177/2047487320916595 | Excluded | Non-data of interest according to BMI |
|  | 2832. Vainshelboim B, Chen Z, Lee YN, et al. Cardiorespiratory Fitness, Adiposity, and Cancer Mortality in Men. Obesity (Silver Spring). 2017;25 Suppl 2:S66-S71. doi:10.1002/oby.22009 | Excluded | Non-data of interest according to PA |
|  | 2843. Van Dam RM, Willett WC, Manson JE, Hu FB. The relationship between overweight in adolescence and premature death in women. Ann Intern Med. 2006;145(2):91-97. doi:10.7326/0003-4819-145-2-200607180-00006 | Excluded | Non-data for outcome of interest |
|  | 2845. van Dam RM, Li T, Spiegelman D, Franco OH, Hu FB. Combined impact of lifestyle factors on mortality: prospective cohort study in US women. BMJ. 2008;337:a1440. doi:10.1136/bmj.a1440 | Excluded | Non-data for outcome of interest |
|  | 2866. Vatten LJ, Nilsen TIL, Romundstad PR, Drøyvold WB, Holmen J. Adiposity and physical activity as predictors of cardiovascular mortality. Eur J Cardiovasc Prev Rehabil Off J Eur Soc Cardiol Work Groups Epidemiol Prev Card Rehabil Exerc Physiol. 2006;13(6):909-915. doi:10.1097/01.hjr.0000239463.80390.52 | Excluded | Non-data of interest according to PA |
|  | 2874. Vest AR, Wu Y, Hachamovitch R, Young JB, Cho L. The Heart Failure Overweight/Obesity Survival Paradox: The Missing Sex Link. JACC Heart Fail. 2015;3(11):917-926. doi:10.1016/j.jchf.2015.06.009 | Excluded | Non-data of interest according to PA |
|  | 2876. Vetrano DL, Collamati A, Magnavita N, et al. Health determinants and survival in nursing home residents in Europe: Results from the SHELTER study. Maturitas. 2018;107:19-25. doi:10.1016/j.maturitas.2017.09.014 | Excluded | Non-data for outcome of interest |
|  | 2930. Wang Y, Wang Y, Qain Y, et al. Association of body mass index with cause specific deaths in Chinese elderly hypertensive patients: Minhang community study. PLoS One. 2013;8(8):e71223. doi:10.1371/journal.pone.0071223 | Excluded | Non-data of interest according to PA |
|  | 2944. Wei M. Cardiorespiratory fitness, adiposity, and mortality [1]. JAMA - J Am Med Assoc. 2008;299(9):1013. doi:10.1001/jama.299.9.1013-a | Excluded | Non-design |
|  | 2945. Wei M, Kampert JB, Barlow CE, et al. Relationship between low cardiorespiratory fitness and mortality in normal-weight, overweight, and obese men. JAMA. 1999;282(16):1547-1553. doi:10.1001/jama.282.16.1547 | Excluded | Non-data of interest according to PA |
|  | 2946. Weinberg CR, Supariwala A, Mian Z, et al. Effect of body mass index on outcome in patients with suspected coronary artery disease referred for stress echocardiography. Am J Cardiol. 2013;112(9):1355-1360. doi:10.1016/j.amjcard.2013.06.024 | Excluded | Non-data of interest according to PA |
|  | 2947. Weinstein AR, Sesso HD, Lee I-M, et al. The joint effects of physical activity and body mass index on coronary heart disease risk in women. Arch Intern Med. 2008;168(8):884-890. doi:10.1001/archinte.168.8.884 | Excluded | Non-data for outcome of interest |
|  | 2952. Wessel TR, Arant CB, Olson MB, et al. Relationship of physical fitness vs body mass index with coronary artery disease and cardiovascular events in women. JAMA. 2004;292(10):1179-1187. doi:10.1001/jama.292.10.1179 | Excluded | Non-data of interest according to PA |
|  | 2957. Whelton SP, McAuley PA, Dardari Z, et al. Association of BMI, Fitness, and Mortality in Patients With Diabetes: Evaluating the Obesity Paradox in the Henry Ford Exercise Testing Project (FIT Project) Cohort. Diabetes Care. 2020;43(3):677-682. doi:10.2337/dc19-1673 | Excluded | Non-data of interest according to PA |
|  | 2982. Williamson DF, Pamuk E, Thun M, Flanders D, Byers T, Heath C. Prospective study of intentional weight loss and mortality in never-smoking overweight US white women aged 40-64 years. Am J Epidemiol. 1995;141(12):1128-1141. doi:10.1093/oxfordjournals.aje.a117386 | Excluded | Non-data for outcome of interest |
|  | 2983. Williamson DF, Pamuk E, Thun M, Flanders D, Byers T, Heath C. Prospective study of intentional weight loss and mortality in overweight white men aged 40-64 years. Am J Epidemiol. 1999;149(6):491-503. doi:10.1093/oxfordjournals.aje.a009843 | Excluded | Non-data of interest according to PA |
|  | 2984. Williamson DF, Thompson TJ, Thun M, Flanders D, Pamuk E, Byers T. Intentional weight loss and mortality among overweight individuals with diabetes. Diabetes Care. 2000;23(10):1499-1504. doi:10.2337/diacare.23.10.1499 | Excluded | Non-data for outcome of interest |
|  | 3005. Woo J, Ho SC, Yuen YK, Yu LM, Lau J. Cardiovascular risk factors and 18-month mortality and morbidity in an elderly Chinese population aged 70 years and over. Gerontology. 1998;44(1):51-55. doi:10.1159/000021983 | Excluded | Non-data for outcome of interest |
|  | 3006. Woo J, Yu R, Yau F. Fitness, fatness and survival in elderly populations. Age (Dordr). 2013;35(3):973-984. doi:10.1007/s11357-012-9398-6 | Excluded | Non-data of interest according to BMI |
|  | 3009. Wu C-Y, Chou Y-C, Huang N, Chou Y-J, Hu H-Y, Li C-P. Association of body mass index with all-cause and cardiovascular disease mortality in the elderly. PLoS One. 2014;9(7):e102589. doi:10.1371/journal.pone.0102589 | Excluded | Non-data for outcome of interest |
|  | 3013. Wu X, Wang W, Zhang D, Zhu F. Nutritional status, lifestyle habits and cancer mortality: a population-based prospective cohort study. Eur J Nutr. Published online November 2021. doi:10.1007/s00394-021-02739-1 | Excluded | Non-data for outcome of interest |
|  | 3014. Wu Z, Huang Z, Wu Y, et al. Sedentary time, metabolic abnormalities, and all-cause mortality after myocardial infarction: A mediation analysis. Eur J Prev Cardiol. 2019;26(1):96-104. doi:10.1177/2047487318804611 | Excluded | Non-data of interest according to BMI |
|  | 3017. Wyse CA, Celis Morales CA, Ward J, et al. Population-level seasonality in cardiovascular mortality, blood pressure, BMI and inflammatory cells in UK biobank. Ann Med. 2018;50(5):410-419. doi:10.1080/07853890.2018.1472389 | Excluded | Non-data for outcome of interest |
|  | 3020. Xiao Q, Keadle SK, Hollenbeck AR, Matthews CE. Sleep duration and total and cause-specific mortality in a large US cohort: interrelationships with physical activity, sedentary behavior, and body mass index. Am J Epidemiol. 2014;180(10):997-1006. doi:10.1093/aje/kwu222 | Excluded | Non-data for outcome of interest |
|  | 3022. Xu H, Zhang M, Xu D, et al. Body mass index and the risk of mortality among Chinese adults with Type 2 diabetes. Diabet Med. 2018;35(11):1562-1570. doi:10.1111/dme.13763 | Excluded | Non-data of interest according to PA |
|  | 3031. Yamashita S, Matsuzawa Y. Adiposity measures and mortality in an Asian population. Nat Rev Endocrinol. 2021;17(2):69-70. doi:10.1038/s41574-020-00446-1 | Excluded | Non-design |
|  | 3032. Yamazaki K, Suzuki E, Yorifuji T, et al. Is there an obesity paradox in the Japanese elderly population? A community-based cohort study of 13 280 men and women. Geriatr Gerontol Int. 2017;17(9):1257-1264. doi:10.1111/ggi.12851 | Excluded | Non-data of interest according to PA |
|  | 3040. Yang YI, Hodge AM, Dugué P-A, et al. Mortality Effects of Hypothetical Interventions on Physical Activity and TV Viewing. Med Sci Sports Exerc. 2021;53(2):316-323. doi:10.1249/MSS.0000000000002479 | Excluded | Non-data for outcome of interest |
|  | 3042. Yang Y, Dugué P-A, Lynch BM, et al. Trajectories of body mass index in adulthood and all-cause and cause-specific mortality in the Melbourne Collaborative Cohort Study. BMJ Open. 2019;9(8):e030078. doi:10.1136/bmjopen-2019-030078 | Excluded | Non-data for outcome of interest |
|  | 3044. Yano C, Kawayama T, Kinoshita T, et al. Overweight improves long-term survival in Japanese patients with asthma. Allergol Int. 2021;70(2):201-207. doi:10.1016/j.alit.2020.09.009 | Excluded | Non-data of interest according to PA |
|  | 3056. Yiengprugsawan V, Banwell C, Zhao J, Seubsman S-A, Sleigh AC. Relationship between body mass index reference and all-cause mortality: Evidence from a large cohort of Thai adults. J Obes. 2014;2014. doi:10.1155/2014/708606 | Excluded | Non-data of interest according to PA |
|  | 3067. Yusuf S, Joseph P, Rangarajan S, et al. Modifiable risk factors, cardiovascular disease, and mortality in 155 722 individuals from 21 high-income, middle-income, and low-income countries (PURE): a prospective cohort study. Lancet (London, England). 2020;395(10226):795-808. doi:10.1016/S0140-6736(19)32008-2 | Excluded | Non-data of interest according to BMI |
|  | 3068. Zafrir B, Salman N, Amir O. Joint impact of body mass index and physical capacity on mortality in patients with systolic heart failure. Am J Cardiol. 2014;113(7):1217-1221. doi:10.1016/j.amjcard.2013.12.030 | Excluded | Non-data of interest according to PA |
|  | 3074. Zaslavsky O, Woods NF, LaCroix AZ, et al. Identification of Risk Factors for Mortality and Poor-Quality-of-Life Survival in Frail Older Women Participating in the Women’s Health Initiative Observational Study. J Am Geriatr Soc. 2016;64(4):831-837. doi:10.1111/jgs.14042 | Excluded | Non-data of interest according to PA |
|  | 3086. Zhang P, Sui X, Hand GA, Hébert JR, Blair SN. Association of changes in fitness and body composition with cancer mortality in men. Med Sci Sports Exerc. 2014;46(7):1366-1374. doi:10.1249/MSS.0000000000000225 | Excluded | Non-data for outcome of interest |
|  | 3100. Zhou Y, Chlebowski R, LaMonte MJ, et al. Body mass index, physical activity, and mortality in women diagnosed with ovarian cancer: results from the Women’s Health Initiative. Gynecol Oncol. 2014;133(1):4-10. doi:10.1016/j.ygyno.2014.01.033 | Excluded | Non-data of interest according to PA |
|  | 3107. Zunzunegui MV, Sanchez MT, Garcia A, Casado JMR, Otero A. Body mass index and long-term mortality in an elderly Mediterranean population. J Aging Health. 2012;24(1):29-47. doi:10.1177/0898264311408419 | Excluded | Non-data for outcome of interest |
|  | Master, H., Annis, J., Huang, S. et al. Association of step counts over time with the risk of chronic disease in the All of Us Research Program. Nat Med 28, 2301–2308 (2022). https://doi.org/10.1038/s41591-022-02012-w | Excluded | Non-population |
|  | Ahmadi, M.N., Lee, IM., Hamer, M. et al. Changes in physical activity and adiposity with all-cause, cardiovascular disease, and cancer mortality. Int J Obes 46, 1849–1858 (2022). https://doi.org/10.1038/s41366-022-01195-z | Excluded | Non-data of interest according to PA |
|  | Heath, L., Jebb, S.A., Aveyard, P. et al. Obesity, metabolic risk and adherence to healthy lifestyle behaviours: prospective cohort study in the UK Biobank. BMC Med 20, 65 (2022). https://doi.org/10.1186/s12916-022-02236-0 | Excluded | Non-data of interest according to PA |
|  | Erik Hemmingsson, Daniel Väisänen, Gunnar Andersson, Peter Wallin, Elin Ekblom-Bak, Combinations of BMI and cardiorespiratory fitness categories: trends between 1995 and 2020 and associations with CVD incidence and mortality and all-cause mortality in 471 216 adults, European Journal of Preventive Cardiology, Volume 29, Issue 6, April 2022, Pages 959–967, https://doi.org/10.1093/eurjpc/zwab169 | Excluded | Non-data for outcome of interest |
|  | Gorzelitz J, Trabert B, Katki HA, et alIndependent and joint associations of weightlifting and aerobic activity with all-cause, cardiovascular disease and cancer mortality in the Prostate, Lung, Colorectal and Ovarian Cancer Screening TrialBritish Journal of Sports Medicine 2022;56:1277-1283. | Excluded | Non-data of interest according to BMI |
|  | He J, Bundy JD, Geng S, Tian L, He H, Li X, Ferdinand KC, Anderson AH, Dorans KS, Vasan RS, Mills KT, Chen J. Social, Behavioral, and Metabolic Risk Factors and Racial Disparities in Cardiovascular Disease Mortality in U.S. Adults : An Observational Study. Ann Intern Med. 2023 Sep;176(9):1200-1208. doi: 10.7326/M23-0507. Epub 2023 Aug 15. PMID: 37579311. | Excluded | Non-data of interest according to BMI |
|  | Harada T, Nagai K, Mase K, Tsunoda R, Iseki K, Moriyama T, Tsuruya K, Fujimoto S, Narita I, Konta T, Kondo M, Kasahara M, Shibagaki Y, Asahi K, Watanabe T, Yamagata K. Elevated Crude Mortality in Obese Chronic Kidney Disease Patients with Loss of Exercise Habit: A Cohort Study of the Japanese General Population. Intern Med. 2023 Aug 1;62(15):2171-2179. doi: 10.2169/internalmedicine.0803-22. Epub 2022 Dec 21. PMID: 36543210; PMCID: PMC10465275. | Excluded | Non-data of interest according to PA |
|  | Cheema BS, Shi Z, White RL, Atlantis E. Associations of Recreational and Nonrecreational Physical Activity and Body Weight Change on Cardiovascular Disease Mortality During the Obesogenic Transition in the United States: National Health and Nutrition Examination Survey Follow-up Study. J Phys Act Health. 2023 Jul 18;20(10):971-979. doi: 10.1123/jpah.2022-0624. PMID: 37463667. | Excluded | Non-data of interest according to BMI |
|  | Webber BJ, Yun HC, Whitfield GP. Leisure-time physical activity and mortality from influenza and pneumonia: a cohort study of 577 909 US adults. Br J Sports Med. 2023 Oct;57(19):1231-1237. doi: 10.1136/bjsports-2022-106644. Epub 2023 May 16. PMID: 37192831. | Excluded | Non-data for outcome of interest |
|  | de Boer WIJ. Do differences in sport participation contribute to socioeconomic health inequalities? Evidence from the Lifelines cohort study on all-cause mortality, diabetes and obesity. Prev Med Rep. 2023 Oct 31;36:102479. doi: 10.1016/j.pmedr.2023.102479. PMID: 37965127; PMCID: PMC10641690. | Excluded | Non-population |
|  | Ferrari G. Lifestyle risk factors and all-cause and cause-specific mortality in the Mexico City prospective study: Assessing the influence of reverse causation. J Affect Disord. 2024 May 1;352:517-524. doi: 10.1016/j.jad.2024.02.072. Epub 2024 Feb 24. PMID: 38408614 | Excluded | Non-data of interest according to BMI |
|  | Rosella LC, Buajitti E. Risk of premature mortality due to smoking, alcohol use, obesity and physical activity varies by income: A population-based cohort study. SSM Popul Health. 2024 Feb 19;25:101638. doi: 10.1016/j.ssmph.2024.101638. PMID: 38426028; PMCID: PMC10904266. | Excluded | Non-data of interest according to BMI |
|  | Zhang Y, Liu X. Effects of physical activity and sedentary behaviors on cardiovascular disease and the risk of all-cause mortality in overweight or obese middle-aged and older adults. Front Public Health. 2024 Feb 12;12:1302783. doi: 10.3389/fpubh.2024.1302783. PMID: 38410660; PMCID: PMC10894908. | Excluded | Non-population |
|  | Ding L. Ideal cardiovascular health and risk of death in a large Swedish cohort. BMC Public Health. 2024 Feb 2;24(1):358. doi: 10.1186/s12889-024-17885-4. PMID: 38308327; PMCID: PMC10837860. | Excluded | Non-population |
|  | Huang Z. Physical Activity and Weight Loss Among Adults With Type 2 Diabetes and Overweight or Obesity: A Post Hoc Analysis of the Look AHEAD Trial. JAMA Netw Open. 2024 Feb 5;7(2):e240219. doi: 10.1001/jamanetworkopen.2024.0219. PMID: 38386318; PMCID: PMC10884882. | Excluded | Non-population |
|  | Sabag A. Timing of Moderate to Vigorous Physical Activity, Mortality, Cardiovascular Disease, and Microvascular Disease in Adults With Obesity. Diabetes Care. 2024 May 1;47(5):890-897. doi: 10.2337/dc23-2448. PMID: 38592034; PMCID: PMC11043226. | Excluded | Non-data of interest according to PA |

| Study | Categories |
| --- | --- |
| Balboa-Castillo T et al., 2011 | Insufficiently active (reference category): participants answering ‘I don’t exercise, my leisure time is spent almost completely in sedentary activities’.  Active: Occasional physical or sports activities, regular physical activity several times a month or physical training several times a week. |
| Crespo et al., 2002 | Insufficiently active (reference category): participants in the quartile 1 after Framingham PA index (index of 27 or less).  Active: participants in quartiles 3-4 (index greater than or equal to 30). |
| de Boer et al., 2021 | Active: Sports participation, as well as the other types of PA assessed from SQUASH questionnaire. Cycling and walking are only considered part of sport participation if they were done as a leisure-time sport discipline.  In this study, we assessed the associations between sport participation (any versus none). |
| Ekelund et al., 2015 | Insufficiently active (reference category): inactive participants, 36 kJ/kg daily.  Active: participants from 46 kJ/kg daily to 51 kJ/kg daily. |
| Min C et al., 2020 | Insufficiently active (reference category): other participants no included in active category.  Active: walking≥5 d/week for ≥30 min, moderate PA ≥5 d/week for ≥30 min or vigorous PA ≥3 d/week for ≥20 min referring to the IPAQ. |
| Patel AV et al., 2010 | Insufficiently active (reference category): <17.5 METs/hour/week. This lowest cutpoint corresponds with approximately the 10th percentile of activity level for the study population.  Active: 17.5 to <24; 24.5 to <31.5;31.5 to <42;42 to <52.5; 52.5 to <63; >=63 METs/hour/week. |
| Sanchez-Lastra M et al., 2021 | Insufficiently active: Low physical activity (first quintile from the IPAQ-SF).  Active: Medium physical activity (quintile 2-3) and High physical activity (quintile 4-5) |
| Wang A et al., 2016 | Insufficiently active: 0 to <100 (inactive)  Active: 500 to <1200 (medium), >1200 (high) METs/min/week. |
| Willey JZ et al., 2015 | Insufficiently active (reference): no active.  Active: Third and fourth quartile of MET-score.  PA was measure with an in-person questionnaire adapted from the National Health Interview Survey of the National Center for Health Statistics of leisure time physical activity (MET-score). |

Table S3. Definitions of physical activity categories (Insufficiently active and active) from original studies.

Figure S1. Forest plot on the effect of physical activity in mortality in adults with overweight.


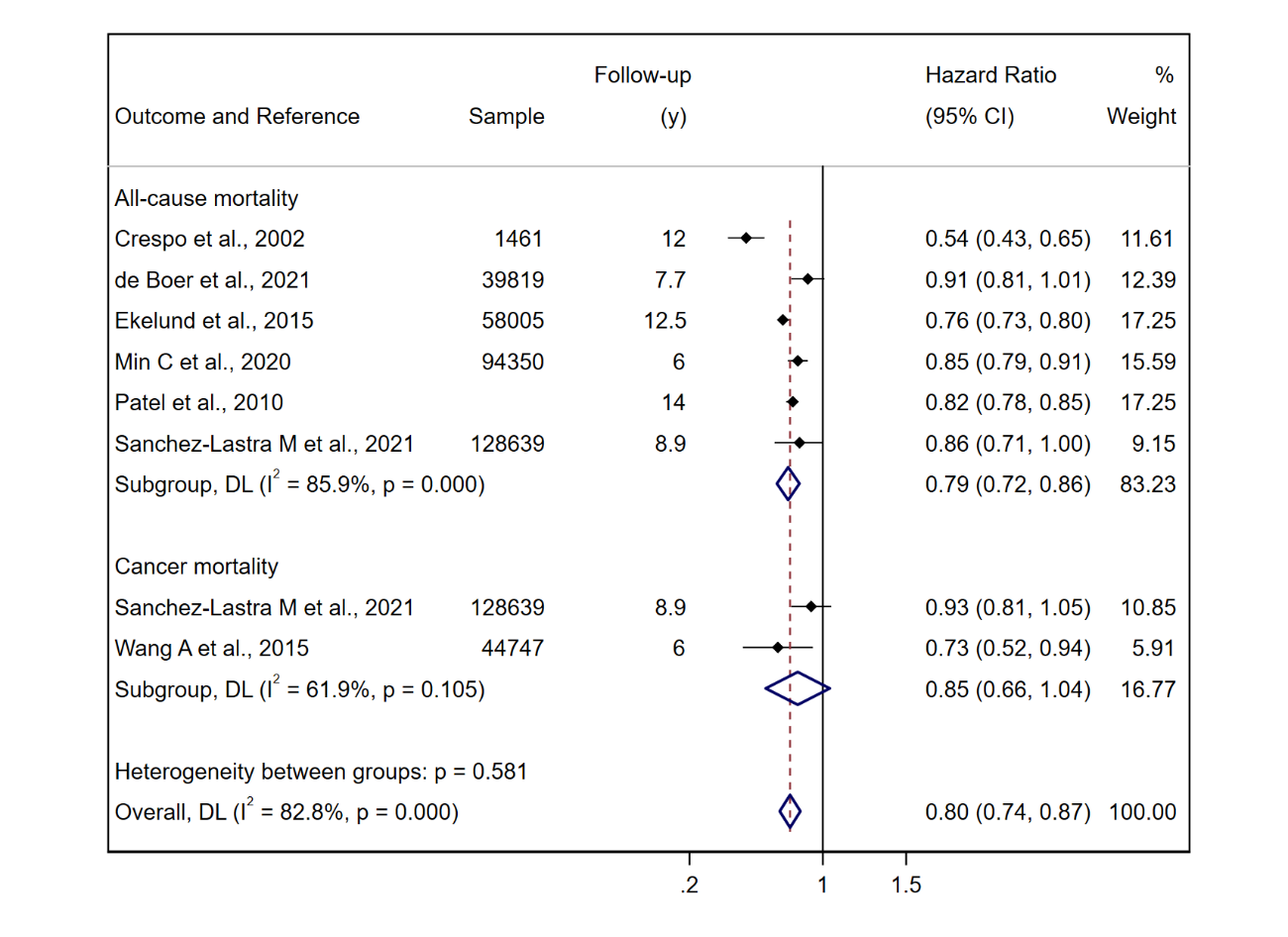


Table S4. The Risk of Bias In Non-randomized Studies – of Exposures (ROBINS-E) assessment tool (for follow-up studies)

| Domains | Balboa-Castillo T et al., 2011 | Crespo et al., 2002 | de Boer et al., 2021 | Ekelund et al., 2015 | Min C et al., 2020 | Patel AV et al., 2010 | Sanchez-Lastra M et al., 2021 | Wang A et al., 2016 | Willey JZ et al., 2015 |
| --- | --- | --- | --- | --- | --- | --- | --- | --- | --- |
| 1.Risk of bias due to confounding | Low Risk of bias | Low Risk of bias | Low Risk of bias | Some concerns | Low Risk of bias | Low Risk of bias | Low Risk of bias | Low Risk of bias | Low Risk of bias |
| 2.Risk of bias arising from measurement of the exposure | Low Risk of bias | Low Risk of bias | Low Risk of bias | Low Risk of bias | Low Risk of bias | Low Risk of bias | Low Risk of bias | Low Risk of bias | Low Risk of bias |
| 3.Risk of bias in selection of participants into the study (or into the analysis) | Low Risk of Bias | Some concerns | Low Risk of bias | Low Risk of Bias | Low Risk of bias | Low Risk of bias | Low Risk of bias | Low Risk of bias | Low Risk of bias |
| 4.Risk of bias due to post-exposure interventions | Los Risk of Bias | Low Risk of bias | Low Risk of bias | Low Risk of bias | Low Risk of bias | Low Risk of bias | Low Risk of bias | Low Risk of bias | Low Risk of bias |
| 5.Risk of bias due to missing data | Low Risk of Bias | Low Risk of bias | Some concerns | Low Risk of bias | Low Risk of bias | Low Risk of bias | Some concerns | Low Risk of bias | Low Risk of bias |
| 6.Risk of bias arising from measurement of the outcome | Low Risk of Bias | Low Risk of bias | Low Risk of bias | Low Risk of bias | Low Risk of bias | Low Risk of bias | Low Risk of bias | Low Risk of bias | Low Risk of bias |
| 7.Risk of bias in selection of the reported result | Low Risk of Bias | Low Risk of bias | Low Risk of bias | Low Risk of bias | Low Risk of bias | Low Risk of bias | Low Risk of bias | Low Risk of bias | Some concerns |
| Overall risk of bias | Low Risk of Bias | Some concerns | Some concerns | Some concerns | Low Risk of Bias | Low Risk of Bias | Some concerns | Low Risk of Bias | Some concerns |

**Judgement Interpretation**

Low risk of bias* there is little or no concern about bias with regard to this domain.

Some concerns there is some concern about bias with regard to this domain, although it is not clear that there is an important risk of bias.

High risk of bias the study has some important problems in this domain: characteristics of the study give rise to a high risk of bias.

Very high risk of bias the study is very problematic in this domain: characteristics of the study give rise to a very high risk of bias.

**Table S5.** GRADE summary of findings.

| **Certainty assessment** | | | | | | | **№ of patients** | | **Effect** | **Certainty** |
| --- | --- | --- | --- | --- | --- | --- | --- | --- | --- | --- |
| **№ of studies** | **Study design** | **Risk of bias** | **Inconsistency** | **Indirectness** | **Imprecision** | **Other considerations** | **Physical activity (moderate-to-high)** | **sedentary** | **Relative (95% CI)** |  |
| 8 | non-randomised studies^a^ | serious^b^ | serious^c^ | serious^d^ | serious^c^ | publication bias strongly suspected strong association all plausible residual confounding would reduce the demonstrated effect dose response gradient^e^ | 84328 participants | 59873 participants | **HR 0.79** (0.74 to 0.84) [All-cause mortality] | ⨁ Very Low |

**CI:** confidence interval; **HR:** hazard Ratio

#### Explanations

a. Prospective cohort studies which means starting from a low level of certainty

b. Failure to adequately control for confounding in observational studies.

c. High heterogeneity as including participants with BMI>30 and differences in physical activity exposures.

d. Most studies were assessed as moderate indirectness, mainly because some studies were not aimed at determining the main effect of physical activity in the specific subgroup of people with obesity.

e. Egger test showed a coefficient of 0.86 (p=0.078).

**Table S6.** Sensitivity analysis for all-cause mortality (n=8).

| **Study omitted** | Hazard Ratio (95% Confidence Interval) |
| --- | --- |
| Balboa-Castillo T et al., 2011 | 0.81 (0.76 to 0.85) |
| Crespo et al., 2002 | 0.80 (0.76 to 0.84) |
| de Boer et al., 2021 | 0.78 (0.73 to 0.84) |
| Ekelund et al., 2015 | 0.78 (0.71 to 0.85) |
| Min C et al., 2020 | 0.79 (0.74 to 0.85) |
| Patel et al., 2010 | 0.77 (0.71 to 0.83) |
| Sanchez-Lastra M et al., 2021 | 0.78 (0.72 to 0.84) |
| Willey JZ et al., 2015 | 0.79 (0.73 to 0.85) |

**Table S7.** Meta-regression models and publication bias for all-cause mortality (n=8).

| **Meta-regressions** |  |  |  |
| --- | --- | --- | --- |
| by follow-up | **Coef** | **SE** | **p** |
|  | 12 | 0.009 | 0.23 |
|  |  |  |  |
| by total sample size | **Coef** | **SE** | **p** |
|  | 1.60 | 1.27 | 0.25 |
|  |  |  |  |
| by physical activity sample | **Coef** | **SE** | **p** |
|  | 4.23 | 2.08 | 0.11 |
|  |  |  |  |
| by sedentary sample | **Coef** | **SE** | **p** |
|  | 3.38 | 3.00 | 0.32 |
|  |  |  |  |
| **Publication bias** | **Coef** | **SE** | **p** |
|  | 0.86 | 0.03 | 0.078 |
